# Knee muscle strength and movement biomechanics in individuals with and without knee pain after anterior cruciate ligament reconstruction: A cross‐sectional study

**DOI:** 10.1002/ksa.12630

**Published:** 2025-02-20

**Authors:** Elisabeth Bandak, Lauri Stenroth, Will Bosch, Kasper Krommes, Johannes Iuel Berg, Henrik Aagaard, Micael Haugegaard, Per Hölmich, Henning Bliddal, Marius Henriksen, Tine Alkjær

**Affiliations:** ^1^ Department of Biomedical Sciences University of Copenhagen Copenhagen Denmark; ^2^ The Parker Institute, Bispebjerg and Frederiksberg Hospital Copenhagen Denmark; ^3^ Department of Technical Physics University of Eastern Finland Kuopio Finland; ^4^ Department of Orthopaedic Surgery, Amager‐Hvidovre, Sports Orthopedic Research Center—Copenhagen (SORC‐C) Copenhagen University Hospital Copenhagen Denmark; ^5^ Ortopaedic Department Zealand University Hospital Koege Koege Denmark; ^6^ Department of Orthopaedic Surgery Gildhøj Private Hospital Copenhagen Denmark

**Keywords:** ACL, biomechanics, knee pain, muscle strength

## Abstract

**Purpose:**

Anterior cruciate ligament injury increases the risk of knee osteoarthritis, possibly via early onset of knee pain and changes in musculoskeletal function. This study compared knee muscle strength and movement biomechanics during walking and forward lunge between individuals with and without knee pain after anterior cruciate ligament reconstruction.

**Methods:**

Cross‐sectional study including participants at least 3 years post anterior cruciate ligament reconstruction, aged 18–40 at the time of surgery, and body mass index ≤30. Symptomatic participants were defined by a knee pain score (reconstructed knee) of ≥3 on a 0–10 scale during activities of daily living in the past week. Asymptomatic participants were defined by a pain score of 0. Maximal isometric quadriceps and hamstring muscle strength (Nm/kg) and 3D walking, and forward lunge movement biomechanics were measured.

**Results:**

A total of 122 participants (30% females) were included: 33 symptomatic and 89 asymptomatic (average age: 33.7, range 23.7–51.3 years). The average post‐surgery time was 6 (range 3–10) years. The symptomatic group exhibited lower isometric quadriceps and hamstring strength with mean group differences (95% confidence interval [CI]) of 0.33 (0.10–0.56) Nm/kg and 0.19 (0.07–0.31) Nm/kg, respectively. There were no important group differences in the walking and forward lunge movement biomechanics.

**Conclusions:**

Symptomatic individuals with anterior cruciate ligament reconstruction demonstrated weaker knee muscles compared to their asymptomatic counterparts. The comparable walking and forward lunge biomechanics suggest that knee pain has no substantial impact on movement biomechanics up to 10 years post‐surgery.

**Level of Evidence:**

Level III.

**Abbreviations**
ACL: anterior cruciate ligament
ADL: activities of daily living
ANCOVA: analysis of covariance
BMI: body mass index
CI: confidence interval
ICOAP: Intermittent and Constant Osteoarthritis Pain questionnaire
IKDC: The International Knee Documentation Committee
KOOS: Knee Injury and Osteoarthritis Outcome Score
MVICs: maximal voluntary isometric contractions
OA: knee osteoarthritis
PDT: pressure pain detection threshold
PTT: pressure pain tolerance threshold
SAP: statistical analysis plan
SD: standard deviation
STROBE: Strengthening the Reporting of Observational Studies in Epidemiology
VRS: verbal rating scale

## INTRODUCTION

Anterior cruciate ligament (ACL) injury significantly impacts lifelong musculoskeletal health due to an increased risk of developing knee osteoarthritis (OA) over time [37, 45]. ACL injuries are common [30, 39], especially in youth [56], often requiring surgical intervention and rehabilitation [17]. Despite these efforts, the risk of knee OA remains high among the ACL reconstructed population [11, 53, 57]. Thus, follow‐up studies have reported that 50% develop OA in their ACL reconstructed knee after 12 years [11]. The aftermath of ACL reconstruction is characterized by changes in musculoskeletal function [53]. Quadriceps muscle deficits, including both reduced strength and impaired activation, are commonly observed after ACL reconstruction [9, 27, 36, 53]. Altered knee joint biomechanics have also been reported, both during normal walking and functional tasks such as forward lunge and hop performance tests [32, 51, 52]. A reduced knee extensor moment during these movements is a characteristic finding following ACL reconstruction [32, 51, 52].

Evidence links impaired musculoskeletal function, especially lower quadriceps muscle strength, to an increased risk of symptomatic and radiographic knee OA [13, 42, 43]. Impaired musculoskeletal function after ACL reconstruction may accelerate OA‐related symptom onset and contribute to the development of OA [13, 43, 51]. However, it is important to stress that the mechanisms by which impaired muscle function contributes to joint degeneration are not fully understood [24]. Poor muscle function may result in impaired micro‐coordination of the knee joint, potentially altering cartilage loading patterns [7, 46, 53]. Altered knee joint loading, whether through overloading or underloading, may negatively impact cartilage health [5, 7, 40, 44]. Notably, one study has associated reduced joint loading during walking with the early onset of knee OA after ACL injury [55]. Thus, the muscle strength and movement biomechanics may change concurrently with the early onset of symptoms, preceding structural OA‐related changes in individuals with ACL reconstruction.

Knee pain is linked to quadriceps weakness and altered movement biomechanics, and notably, it has been observed to affect knee joint biomechanics [22, 23, 48]. However, the specific impact of knee pain on muscle strength and movement biomechanics following ACL reconstruction remains incompletely understood. Furthermore, knee pain is a predictor for developing knee OA [41]. Investigating differences in muscle strength and movement biomechanics between individuals with and without knee pain after ACL reconstruction may enhance our understanding of factors that increase the risk of accelerated knee OA development in this population. Assessment of movement biomechanics could reasonably include walking, given its fundamental importance in daily life and the high number of steps taken each day. To supplement this with a more demanding movement task for the knee joint, the forward lunge has been shown to be suitable due to its discriminatory power, acceptable reliability, and feasibility for the ACL reconstructed population [1, 2, 3, 4, 21, 52]. Thus, we aimed to compare knee muscle strength and movement biomechanics (walking/forward lunge) between individuals with (symptomatic) and without (asymptomatic) knee pain at least 3 years after ACL reconstruction. In addition, we assessed self‐reported knee function, current knee pain during testing, activity level, and pressure pain sensitivity to describe group differences in these parameters. We hypothesized that symptomatic individuals with ACL reconstruction have weaker quadriceps muscles and develop lower muscle forces and knee joint loading during walking and forward lunge compared to their asymptomatic counterparts.

## METHODS

### Study design and setting

This cross‐sectional study, conducted at The Parker Institute, Bispebjerg and Frederiksberg Hospital, Copenhagen, Denmark, adhered to the Declaration of Helsinki and received approval from the health research ethics committee of the Capital Region of Denmark (H‐20060332). The study protocol, which is accessible in the online supplemental material, was developed and made publicly available on The Parker Institute's website (http://www.parkerinst.dk/ongoing-projects/musculoskeletal-function-anterior-cruciate-ligament-reconstructed-individuals-and) prior to partcipant inclusion. The manuscript adheres to the Strengthening the Reporting of Observational Studies in Epidemiology (STROBE) guidelines [15].

### Participants

We identified participants who underwent ACL reconstruction at designated hospitals from 2013 to 2019 via the Danish Ligament Reconstruction Register [35]. This timeframe was chosen to balance the need for an adequate sample with minimizing the inclusion of participants with a long time since surgery. Invitations were dispatched electronically. The recruitment period ran from June 2021 and June 2022. Interested respondents underwent a telephone pre‐screening before receiving an invitation to a clinical screening examination for inclusion purposes.

Eligible participants were aged 18–40 years at the time of primary ACL reconstruction (semitendinosus‐gracilis tendon graft, single‐bundle), with a post‐surgery time of at least 3 years and a current body mass index (BMI) of ≤30. Symptomatic participants were defined by an average knee pain score of at least 3 on a 0–10 verbal rating scale (VRS) in the reconstructed knee during activities of daily living (ADL) in the past week, while asymptomatic participants were defined by a pain score of 0. Pain levels of 1 or 2 were considered minimal and unlikely to interfere with daily life, based on clinical experience, and were excluded to enhance the distinction between symptomatic and asymptomatic groups.

Major exclusion criteria included known neuromuscular diseases, severe cartilage lesions at the time of injury, surgery on the contralateral knee, and musculoskeletal pain beyond the injured knee. All participants provided written informed consent prior to participating in any study‐related procedures.

### Sample size

Our sample size estimation was based on the detection of a difference in the quadriceps muscle strength (primary outcome) between symptomatic and asymptomatic individuals with ACL reconstruction aiming for a 1:1 group allocation. Due to unknown variance within the population, our sample size estimation was based on pragmatic considerations, informed by existing literature, including quadriceps strength deficits in ACL reconstructed populations [36]. To detect a group difference of 0.3 Nm/kg in the primary outcome with a common standard deviation of 0.5 Nm/kg, a total of *n* = 100 sample size with a size of 50 per group resulted in a power of 84% (*α* = 0.05). The standard deviation of 0.5 Nm/kg was selected to allow for the detection of a moderate effect size of 0.6.

Six months after study initiation, 71 participants (17/54 symptomatic/asymptomatic) were recruited, reflecting a low prevalence of symptomatic participants. We anticipated difficulties in recruiting 50 symptomatic participants within the study timeframe and decided to change the group distribution ratio from 1:1 to 1:3. A re‐calculation of the sample size using the same parameters as stated above resulted in a new total sample size of *n* = 120 (*n* = 30 symptomatic and *n* = 90 asymptomatic participants) with a statistical power of 80.6%. All details regarding our sample size estimation are provided in the study protocol and statistical analysis plan (SAP) available in the online supplemental material.

### Outcome measures

All outcomes were prespecified and obtained from the participants' ACL reconstructed knee. The primary outcome was maximal isometric quadriceps muscle strength. Key secondary outcomes were peak extensor moment, peak quadriceps muscle force, and peak total knee joint contact force, assessed during walking and forward lunge. All additional outcomes described in the following were classified as other secondary outcomes.

### Muscle strength and movement biomechanics

Isometric quadriceps and hamstring muscle strength were assessed using an isokinetic dynamometer (Biodex System4 Pro; Biodex Medical System). Prior to the tests, the participants had a 10‐min warm‐up on a stationary bike. Following, the participants sat in a rigid chair and were firmly strapped to the seat at the hip, torso, and distal thigh. The rotation axis of the dynamometer was visually aligned to the lateral femoral epicondyle, and the lower leg was attached to the lever arm of the dynamometer. The lever arm was placed two finger widths above the lateral malleolus and fixed with a cuff. Maximal voluntary isometric contractions (MVICs) of the quadriceps and hamstring muscles were assessed at 60° knee flexion. Visual real‐time feedback on a computer screen and loud verbal encouragement from the tester was provided during testing. The participants were instructed to habituate to the test by practicing isometric contractions of both knee extensors and flexors at submaximal levels. Following this, three maximal knee extension and flexion contractions were performed in an alternating fashion. Each MVIC lasted for 5 s with 30 s resting time in between. The maximal isometric strength of both the quadriceps and hamstring muscles was determined as the highest torque values measured among the three separate MVIC repetitions for each muscle group. These values were normalized to body mass and are expressed in Nm/kg.

Walking and forward lunge biomechanics were assessed in a motion capture laboratory equipped with 12 infrared cameras and two force plates using standard three‐dimensional motion capture software (Vicon MX, Vicon Motion Systems, Vicon Nexus version 2.12). In total, 39 small retroreflective markers were attached to the participant's skin over well‐defined anatomical landmarks according to an extended version of the lower body Conventional Gait Model 2.4 [12] with additional markers on the torso (Supporting Information S1: Figure S1). Marker trajectories and force plate data were recorded at 100 and 1000 Hz, respectively.

Walking tests were performed at self‐selected speed (target speed) on a 10 m long walkway with two ground‐embedded force plates located midway of the walkway (AMTI OR 6‐5‐1000). Participants were instructed to walk in a natural manner, simulating a comfortable stroll on a sidewalk, while maintaining a forward gaze. After a few familiarization trials and target speed determination, the participants performed enough walking tests to capture six trials with clean force plate contacts for both legs all within ±0.1 km/h of the target speed.

The forward lunge movements were performed by taking one step forward, placing the leading foot on the force plate, flexing the knee to approximately 90° and subsequently returning to the starting posture as fast as possible by pushing off from the ground. Participants were instructed to have their hands on the back of the head, the upper body perpendicular to the ground, and the opposite foot maintaining ground contact during the movement. After a few habituation trials, three forward lunges were performed and captured.

Musculoskeletal modelling and simulation software OpenSim (version 4.1) [49] was used to analyse the walking and forward lunge motion capture data which included quantification of kinematics (knee joint angle) and kinetics (e.g., joint moments) during the stance/contact phase of walking/forward lunge of which more details are presented in the online supplementary material. Subsequently, the following outcomes were extracted: peak knee flexion angle, peak knee extensor moment, peak quadriceps muscle force and peak total knee (tibiofemoral) joint contact force. These outcomes were extracted from the first half of the stance phase (walking) and the entire foot‐ground contact phase (forward lunge). In addition, walking speed and forward lunge foot‐ground contact time were extracted. Means of the trials selected for analysis were calculated for each variable. All moments and forces were normalized to body mass (i.e., Nm/kg and N/kg).

Current knee pain on the reconstructed side was assessed using VRS 0–10, with 0 indicating ‘no pain at all’ and 10 ‘worst imaginable pain’ immediately after each of the MVICs during the strength testing and each walking/forward lunge trial and calculated as the mean VRS reported during the trials selected for analysis.

### Pressure pain sensitivity

DoloCuff, a computerized cuff pressure algometry device with software version 2.0.5.1 (DoloCuff, Unique Electronic Aps) was used to assess pressure pain sensitivity [28]. A double‐chambered Tourniquet cuff was wrapped around the gastrocnemius muscles of the ACL reconstructed leg. The cuff was inflated with air at 1 kPa/s by a computer‐controlled compressor [26]. The participants used a handheld device to record their pressure pain detection threshold (PDT) and later pressed the stop button to indicate the pressure pain tolerance threshold (PTT). The test was done four times, each separated by 3‐minute resting periods. The initial measurement served for familiarization, while the subsequent three were stored for further analysis. The two pressure pain sensitivity thresholds (PDT, PTT) were both calculated as means of the three measurements and expressed in kPa.

### Self‐reported knee function and activity level

Self‐reported knee pain/function was assessed by the participants completing the following questionnaires: The International Knee Documentation Committee (IKDC) [25], the Knee Injury and Osteoarthritis Outcome Score (KOOS) [47], and the Intermittent and Constant Osteoarthritis Pain questionnaire (ICOAP) [20]. The IKDC questionnaire, including three subscales: symptoms, sports activity, and knee function, was converted into an overall function score, ranging from 0 to 100, representing the level of function (worst to best) [25]. The KOOS questionnaire consists of five individual KOOS subscales: pain, symptoms, function in ADL, function in sport and recreation, and knee‐related quality of life, each calculated as a normalized score ranging from 0 to 100 (worst to best) [47]. The ICOAP questionnaire [20] includes two subscales: constant pain and intermittent pain, each scored on a 0–100 scale (best to worst). Additionally, a total pain score was derived by summing the ICOAP constant and intermittent pain subscales, also resulting in a 0–100 score (best to worst).

The Tegner score [54] was utilized to assess activity levels with the participants reporting the extent of their engagement that best described both their pre‐injury and current activity levels. The Tegner score rates activity levels from 0 (complete disability) to 10 (highly competitive sports participation) [54]. Pre‐injury (based on recall) and current Tegner scores were recorded for each participant, and the change in score was calculated.

### Demographic variables

Demographic information was recorded either during telephone pre‐screening (type of sport, return to sport) or at the measurement visit (age, sex, height, body mass, BMI, injured knee, time since surgery, knee joint laxity, activity level and radiographic knee OA grade). Bilateral standing knee radiographs were obtained and evaluated by an experienced rheumatologist (H.B.) to determine the radiographic knee OA level using the Kellgren–Lawrence grading [31]. The Kellgren–Lawrence scores range from 0 to 4, with scores of 2, 3, or 4 indicating definite radiographic OA and higher scores indicating more severe changes.

Instrumented knee joint laxity testing quantifying the anterior translation of the tibia relative to the femur was measured using a digital arthrometer (Lachmeter, Lachmeter Company Equipamentos Ortopédicos LTDA). The laxity measurements were done three times for both knees. The mean of the three instrumented knee joint laxity measurements was calculated for both knees and then used to calculate the side‐to‐side difference in millimetres by subtracting the knee joint laxity of the contralateral knee from that of the ACL reconstructed knee.

### Statistical analysis

The analysis was performed according to the SAP (online supplemental material) that was publicly available online (http://www.parkerinst.dk/ongoing-projects/statistical-analysis-plan-mirakos) before conducting any data analyses.

The primary analysis applied for the estimation of between‐group (symptomatic versus asymptomatic) differences of all selected outcomes was an analysis of covariance (ANCOVA) for continuous data. The results are reported as mean ± standard deviation (SD), mean differences with 95% confidence interval (CI). Categorical data and counts (percentages) were analysed using chi‐square statistics comparing distributions between groups. The level of significance was set to 0.05. The analyses were done using the statistical software SAS version 9.4 (SAS Institute Inc.).

## RESULTS

### Participants and demographics

**Figure 1:**
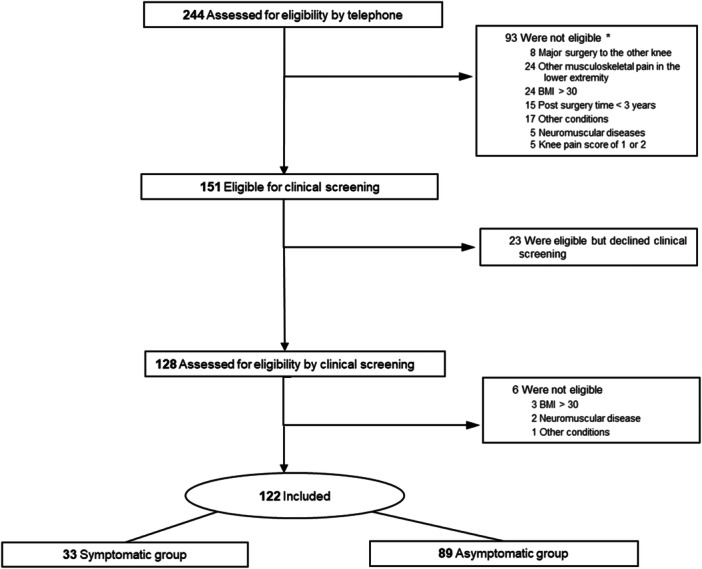
Flow diagram illustrating the inclusion and exclusion of study participants from initial telephone pre‐screening to the final analysis. Numbers at each stage indicate the count of participants, and reasons for exclusions. *Total counts may exceed the number of excluded subjects as they may have multiple exclusion reasons. BMI, body mass index.

A total of 738 invitations were sent to potential participants identified in the Danish Ligament Reconstruction Register of which 244 (33%) responded and underwent telephone pre‐screening (Figure 1). After clinical screening, 122 eligible participants (30% females) were included, of which 33 were identified as symptomatic and 89 as asymptomatic. During the telephone pre‐screening, five potential participants reported knee pain scores of 1 or 2 in their reconstructed knee during ADL in the past week. These five subjects were found ineligible due to other exclusion criteria. Median knee pain scores during ADL in the past week at inclusion for the symptomatic group was 3 (range 3–7). The average time since ACL reconstruction was approximately 75 months (6 years, range 3–10) for both the symptomatic and asymptomatic groups (Table 1). Seventy‐five percent of all participants had a post‐surgery time of less than 8 years, while 25% had a post‐surgery time exceeding 8 years. Most of the demographic characteristics were comparable for both groups except for the frequency of return to sports, which was lower for the symptomatic group compared to the asymptomatic group (Table 1). Among all participants, 70.5% exhibited no radiographic signs of OA, while 23% had Kellgren–Lawrence scores of 2, and 6.5% had scores exceeding 2 (Table 1).

**Table 1:** Participant characteristics.

	Asymptomatic (*N* = 89)	Symptomatic (*N* = 33)	Estimated difference	*p*‐Value
Characteristics				
Age, years	34.0 (6.9)	32.8 (6.6)	1.3 (−1.5 to 4.0)	0.369
Male sex, no. (%)	64 (71.9)	21 (63.6)	‐	0.377
Height, m	1.79 (0.09)	1.77 (0.09)	0.02 (−0.02 to 0.05)	0.308
Body mass, kg	78.8 (11.7)	80.2 (11.5)	−1.3 (−6.0 to 3.4)	0.573
Body mass index, kg/m^2^	24.6 (2.5)	25.6 (2.7)	−1.0 (−1.9 to 0.1)	0.066
Injured knee (right), no. (%)	48 (53.9)	16 (48.5)	‐	0.593
Time since surgery, month	75.8 (20.5)	75.1 (19.1)	0.7 (−7.4 to 8.8)	0.862
Knee joint laxity, mm	0.91 (1.70)	1.25 (1.91)	−0.34 (−1.05 to 0.37)	0.342
Injury situation,^a^ no. (%)				
Traffic accident	0 (0)	0 (0)	‐	
Sport injury	81 (91.0)	28 (84.8)	‐	0.327
Other	8 (9.0)	5 (15.2)	‐	
Type of sport,^a^ no. (%)				
Team ball sports	61 (75.3)	20 (71.4)	‐	
Skiing	13 (16.1)	3 (10.7)	‐	
Racket sports	4 (4.9)	1 (3.6)	‐	0.354
Martial arts	2 (2.5)	2 (7.1)	‐	
Other	1 (1.2)	2 (7.1)	‐	
Return to sport,^a^ no. (%)^b^				
No	29 (33.7)	19 (61.3)	‐	
Yes	47 (54.7)	6 (19.4)	‐	*0.003*
Partly^d^	10 (11.6)	6 (19.4)	‐	
Kellgren–Lawrence score,^c^ no. (%)				
0	37 (41.6)	14 (42.4)	‐	
1	25 (28.1)	10 (30.3)	‐	
2	20 (22.5)	8 (24.2)	‐	0.906
3	6 (6.7)	1 (3.0)	‐	
4	1 (1.1)	0 (0.0)	‐	
Activity level				
Pre‐injury activity level (Tegner score)	4.1 (1.8)	4.2 (1.5)	−0.1 (−0.7 to 0.6)	0.816
Current activity level (Tegner score)	5.6 (1.4)	5.8 (1.6)	−0.3 (−0.8 to 0.3)	0.358

*Note*: Values are means (SD) if nothing else stated. Estimated differences are presented as group mean differences (95% confidence interval) for continuous data.

^a^ Obtained during screening interview.

^b^ Missing answers: 3 (asymptomatic), 2 (symptomatic).

^c^ Scores on the Kellgren–Lawrence scale range from 0 to 4, with a score of 2, 3 or 4 indicating definite osteoarthritis and higher scores indicating more severe disease.

^d^ Meaning ‘yes, but not at the same pre‐injury level’.

### Muscle strength, movement biomechanics and pressure pain sensitivity

The symptomatic group had lower isometric muscle strength compared to the asymptomatic group (Table 2). On average, the symptomatic group exhibited a 12% lower quadriceps muscle strength and a 15% lower hamstring muscle strength compared to the asymptomatic group.

**Table 2:** Group means (SD) and estimated group mean differences (95% confidence interval [CI]) of muscle strength, walking and forward lunge knee biomechanics, knee pain during movement/muscle strength tests and pressure pain sensitivity variables including statistical probability.

	Asymptomatic (*N* = 89)	Symptomatic (*N* = 33)	Estimated difference	
	Mean (SD)	Mean (SD)	Group mean difference (95% CI)	*p*‐Value
Muscle strength				
Maximal isometric quadriceps muscle strength (Nm/kg)^a^	2.76 (0.57)	2.43 (0.56)	0.33 (0.10 to 0.56)	0.0045
Maximal isometric hamstring muscle strength (Nm/kg)^b^	1.30 (0.30)	1.11 (0.27)	0.19 (0.07 to 0.31)	0.0016
Walking biomechanics				
Peak knee extensor moment (Nm/kg)^c^	0.80 (0.26)	0.82 (0.22)	−0.02 (−0.12 to 0.09)	0.7558
Peak quadriceps muscle force (N/kg)^c^	16.38 (4.22)	16.68 (4.38)	−0.29 (−2.02 to 1.43)	0.7350
Peak knee joint contact force (N/kg)^c^	29.87 (3.84)	29.93 (4.47)	−0.07 (−1.69 to 1.56)	0.9353
Peak knee flexion (°)^b^	21.3 (5.2)	22.6 (4.0)	−1.3 (−3.3 to 0.6)	0.1818
Walking speed (m/s)^b^	1.33 (0.15)	1.33 (0.18)	−0.00 (−0.67 to 0.06)	0.9222
Forward lunge biomechanics				
Peak knee extensor moment (Nm/kg)^c^	1.56 (0.25)	1.47 (0.27)	0.09 (−0.02 to 0.19)	0.1062
Peak quadriceps muscle force (N/kg)^c^	48.07 (6.86)	44.75 (7.69)	3.32 (0.46 to 6.18)	0.0233
Peak knee joint contact force (N/kg)^c^	55.10 (13.05)	51.17 (10.02)	3.93 (−1.04 to 8.90)	0.1202
Peak knee flexion (°)^b^	106.6 (7.5)	103.8 (8.9)	2.8 (−0.4 to 6.0)	0.0844
Forward lunge foot‐ground contact time (s)^b^	1.17 (0.24)	1.27 (0.36)	−0.10 (−0.21 to 0.02)	0.0901
Pain during movement/muscle strength tests				
Current knee pain, quadriceps muscle strength test^b^	0.45 (0.98)	1.20 (1.43)	−0.75 (−1.20 to −0.30)	0.0013
Current knee pain, hamstring muscle strength test^b^	0.12 (0.41)	0.35 (0.66)	−0.23 (−0.43 to −0.04)	0.0202
Current knee pain, walking^b^	0.12 (0.62)	0.11 (0.39)	0.01 (−0.22 to 0.24)	0.9372
Current knee pain, forward lunge^b^	0.17 (0.70)	0.76 (1.18)	−0.59 (−0.94 to −0.25)	0.0010
Pressure pain sensitivity				
Pressure pain detection threshold (kPa)^b^	27.5 (16.2)	24.4 (11.9)	3.1 (−3.1 to 9.2)	0.3249
Pressure pain tolerance threshold (kPa)^b^	71.8 (24.6)	69.9 (25.1)	1.9 (−8.0 to 11.9)	0.7006

^a^ Primary outcome measure.

^b^ Other secondary outcome measures.

^c^ Key secondary outcome measures.

The movement biomechanics were generally consistent across groups, except for a lower peak quadriceps muscle force observed during forward lunge in the symptomatic group compared to the asymptomatic group (Table 2).

In general, both groups reported low average knee pain levels (i.e., <1) during the tests, with the symptomatic group reporting slightly higher pain levels during muscle strength and forward lunge testing than the asymptomatic group (Table 2).

No group differences were observed in any of the pressure pain sensitivity thresholds (Table 2).

### Self‐reported knee function and activity level

Overall, the self‐reported knee function and pain score results showed that the symptomatic group was more impaired than the asymptomatic group (Table 3). One exception was the ICOAP constant subscale, which did not differ between the groups. Furthermore, the change in Tegner score from pre‐injury to current state was similar between the groups (Table 3).

**Table 3:** Group means (SD) and estimated group mean differences (95% confidence interval [CI]) of questionnaires (patient‐reported outcomes) and activity level variables, including statistical probability.

	Asymptomatic (*N* = 89)	Symptomatic (*N* = 33)	Estimated difference	
	Mean (SD)	Mean (SD)	Group mean difference (95% CI)	*p*‐Value
*Questionnaires* a				
KOOS Pain score	93.3 (10.7)	77.2 (10.2)	16.1 (11.8 to 20.3)	<0.0001
KOOS Symptoms score	65.9 (10.8)	55.6 (9.6)	9.2 (5.0 to 13.5)	<0.0001
KOOS Quality of life score	74.2 (18.2)	52.1 (14.2)	22.1 (15.1 to 29.0)	<0.0001
KOOS Function in sport and recreation score	83.7 (15.8)	57.0 (16.4)	26.7 (20.3 to 33.2)	<0.0001
KOOS Function in ADL score	96.9 (7.7)	85.7 (11.0)	11.3 (7.8 to 14.8)	<0.0001
IKDC score	86.2 (11.0)	64.5 (10.7)	21.7 (17.3 to 26.2)	<0.0001
ICOAP Total score	3.0 (9.5)	16.9 (9.2)	−13.9 (−17.7 to −10.1)	<0.0001
ICOAP Constant pain subscore	1.1 (8.6)	3.3 (8.7)	−2.2 (−5.7 to 1.3)	0.2110
ICOAP Intermittent pain subscore	4.6 (11.6)	28.3 (14.3)	−23.6 (−28.7 to −18.6)	<0.0001
Activity level^a^				
Change in Tegner score^b^	1.4 (1.7)	1.6 (1.6)	−0.2 (−0.9 to 0.5)	0.5844

Abbreviations: ADL, activities of daily living; ICOAP, Intermittent and Constant Osteoarthritis Pain; IKDC, The International Knee Documentation Committee; KOOS, Knee Injury and Osteoarthritis Outcome Score.

^a^ Other secondary outcome measures.

^b^ The change in Tegner score is the difference between the current and pre‐injury Tegner scores.

## DISCUSSION

We found that, on average 6 years after ACL reconstruction, individuals with knee pain had weaker quadriceps and hamstring muscles than their asymptomatic counterparts but seemed to employ similar walking and forward lunge biomechanics. The quadriceps muscle strength deficit in the symptomatic group aligned with our hypothesis, while the movement biomechanics results contradicted our hypothesis and previous assertions that knee pain is associated with decreased knee joint loading during functional movements [48].

The knee muscle weakness observed among our symptomatic participants may be caused by both morphological and neuromuscular factors [9, 36, 48, 50, 53]. The presence of pain itself has the potential to constrain muscle activation [23, 48], suggesting that it could contribute to the strength deficits in the symptomatic group. The symptomatic group reported higher levels of current knee pain during both muscle strength and movement biomechanics testing, but these levels were very low. This suggests that the knee pain during the tests may not have been a substantial limiting factor on muscle strength and movement biomechanics. However, the influence of even low pain levels on the measurements remains unclear.

Muscle weakness is associated with an increased risk of development of both symptomatic and radiographic knee OA [13, 33, 43]. Further, the presence of knee pain also associates with an increased risk of knee OA development [41]. Therefore, the combination of knee pain and knee muscle weakness in our symptomatic participants may place them at a higher risk of developing knee OA compared to those without knee pain. While the implications of muscle strength in relation to the onset and progression of knee OA are not fully understood [33], studies suggest that both quadriceps and hamstring strength training are associated with alleviating knee pain symptoms [18, 29, 33]. In relation to the ACL reconstructed population, a recent study revealed that supervised hamstring strength training had a positive impact on knee flexor strength and alleviated knee pain in individuals with hamstring strength deficits following ACL reconstruction [10]. Thus, it seems relevant for a future study to test if targeted strength training of the quadriceps and hamstrings can reduce knee pain in symptomatic individuals with ACL reconstruction. Additionally, the impact of interventions on long‐term knee joint health in the ACL reconstructed population should be investigated. This involves potential mitigation of structural joint degeneration and symptomatic/functional impairments.

Our findings suggest that movement biomechanics are largely unaffected by knee pain experienced during daily living in young individuals with ACL reconstruction with a post‐surgical time of 3–10 years. Despite observing a lower peak quadriceps muscle force during forward lunge in our symptomatic group, this does not conclusively suggest an impact on their overall movement biomechanics. The observation that movement biomechanics did not differ although knee muscle strength differed between the groups aligns with previous studies reporting no clear correlation between knee extensor strength and knee joint movement biomechanics [14, 52]. It is possible that the walking and forward lunge movements applied in our study are too submaximal to be impacted by the muscle strength level. Thus, the movement biomechanics assessments included in the current study do not seem to help identify individuals at higher risk of developing early onset knee OA after ACL surgery.

Overall, the asymptomatic and symptomatic groups were comparable across most of the demographic variables except for a lower return to sport frequency in the symptomatic group. However, the reported current activity levels (Tegner scores) were comparable between the symptomatic and asymptomatic groups, indicating an active lifestyle involving moderate engagement in recreational activities and sports. In contrast, the self‐reported knee function was lower in the symptomatic group than the asymptomatic group, underlining the distinction between them. The mean IKDC score of ~65 for the symptomatic group corresponds to a lower score than 85%–90% of the normative reference population between the ages of 25 and 34 years [6]. Furthermore, four out of five KOOS subscales levels were below 85, indicating that our symptomatic group had significant knee symptoms, potentially reflecting early signs knee OA illness [16, 19]. This is further supported by the ICOAP results, which showed that the symptomatic group experiences intermittent rather than constant knee pain. An intermittent pain pattern is characteristic of early‐stage knee OA and is often observed with limited or no radiographic changes [38].

The pressure pain thresholds were similar across the symptomatic and asymptomatic groups and resembled values reported for healthy young individuals aged between 20 and 29 years [34]. While individuals with chronic musculoskeletal pain, such as knee OA [8] often exhibit increased pain sensitivity, potentially resulting from central sensitization, our findings did not indicate sensitization of the nociceptive system in the symptomatic group.

Our study has limitations requiring further consideration. First, while we aimed to test hypotheses, it is important to acknowledge that the cross‐sectional study design renders our analyses exploratory in nature. Second, our group stratification, relying on the absence or presence of knee pain during ADL within the past week, may have led to a few participants being misclassified due to variations in lifestyle and/or pain‐coping strategies. However, the clear contrast in self‐reported knee function and pain scoring between the two groups suggests limited misclassification. Third, our statistical analyses included multiple comparisons without adjustment for multiplicity, which may be critical for the movement biomechanics due to the several outcomes involved, increasing the likelihood of chance findings. Hence, the sole statistically significant outcome (the peak quadriceps muscle force during forward lunge) observed among all movement biomechanics measures is considered unimportant. Moreover, while no adjustments for confounding covariates were made in our analyses, we acknowledge the potential influence of measured and unmeasured variables on our results. However, we have not identified any measured covariates as potential confounders. Additionally, we lacked information on postoperative rehabilitation protocols and concurrent surgical procedures such as meniscectomy or meniscus repair. While such information could offer a more detailed description of our study sample, it is uncertain if it can have an impact on long‐term changes in muscle strength and movement biomechanics. Furthermore, we did not collect detailed data on the location of participants' knee pain, as they were asked to report general knee pain rather than region‐specific pain. This limits our ability to discern whether the reported pain is specific to certain knee joint regions, such as the patellofemoral or other compartments. Finally, the findings may not generalize to individuals who have undergone other types of ACL reconstruction grafts than a semitendinosus‐gracilis tendon or individuals with concomitant severe damage to knee joint cartilage or other joint structures at the time of injury.

In conclusion, our findings showed that 3–10 years after ACL reconstruction, individuals with knee pain exhibited weaker quadriceps and hamstring muscles compared to their asymptomatic counterparts. In contrast, walking and forward lunge movement biomechanics were comparable across groups suggesting that knee pain has no substantial impact on movement biomechanics up to 10 years after ACL reconstruction.

## AUTHOR CONTRIBUTIONS

Elisabeth Bandak and Tine Alkjær had full access to all data of the study and are responsible for the data and the analyses. *Concept and design*: Tine Alkjær, Marius Henriksen, Henning Bliddal, Per Hölmich, Lauri Stenroth, Henrik Aagaard and Elisabeth Bandak conceived and designed the study and protocol. *Obtained funding*: Tine Alkjær, Lauri Stenroth, Marius Henriksen, Elisabeth Bandak and Henning Bliddal. *Statistical analysis*: Tine Alkjær, Elisabeth Bandak and Marius Henriksen. *Drafting of the manuscript*: Elisabeth Bandak and Tine Alkjær. *Acquisition, analysis or interpretation of data, and critical revision of the manuscript for important intellectual content*: All authors.

## CONFLICTS OF INTEREST STATEMENT

Marius Henriksen is an Associate Editor of Osteoarthritis and Cartilage, is on the scientific advisory board of the Thuasne group; and has received travel grants from Contura International A/S. The remaining authors declare no conflicts of interest.

## ETHICS STATEMENT

The study adhered to the Declaration of Helsinki and received approval from the health research ethics committee of the Capital Region of Denmark (H‐20060332). All participants provided written informed consent prior to participating in any study‐related procedures.

## REGISTRATION OF STUDY PROTOCOL AND STATISTICAL ANALYSIS PLAN

The study protocol was developed and approved before study initiation and inclusion of any participants. The protocol was made publicly available at the Parker Institute's website (https://www.parkerinst.dk/ongoing-projects/musculoskeletal-function-anterior-cruciate-ligament-reconstructed-individuals-and). The protocol is also included in the Supporting Information.

Likewise, the statistical analysis plan (SAP) was developed and made publicly available (https://www.parkerinst.dk/ongoing-projects/statistical-analysis-plan-mirakos) before conducting analyses. The SAP is also included in the Supporting Information. These information is also given in the manuscript but hidden in the submitted manuscript text to ensure anonymity.

## Supporting information

Supporting information.

Supporting information.

## Data Availability

The data are available from the corresponding author upon reasonable request.
