# Supplementary material for: Knee muscle strength and movement biomechanics in individuals with and without knee pain after anterior cruciate ligament reconstruction: A cross‐sectional study
Source: Knee Surg Sports Traumatol Arthrosc. 2025 Feb 20;33(12):4136–47. doi: 10.1002/ksa.12630 (PMC12684341; doi:10.1002/ksa.12630)
Supplement: Supplementary file 1 — Supporting information. [file KSA-33-4136-s002.pdf]

STROBE Statement—Checklist of items that should be included in reports of *cross-sectional studies.*

	Item No	Recommendation	Page No.	Relevant text from the manuscript
**Title and abstract**	1	(*a*) Indicate the study's design with a commonly used term in the title or the abstract	1+2	The cross-sectional study design is explicitly stated in both the title and abstract. Example from the abstract: "Methods: Cross-sectional study including participants at…".
		(*b*) Provide in the abstract an informative and balanced summary of what was done and what was found	2	Provided in the abstract.
**Introduction**				
Background/rationale	2	Explain the scientific background and rationale for the investigation being reported	3	ACL injury, quadriceps muscle weakness and knee pain are all knee OA risk factors. Little is known about how muscle function develops after ACL reconstruction and if it differs between those with and without knee pain. This is clearly described in the introduction.
Objectives	3	State specific objectives, including any prespecified hypotheses	4	Lines 69-75: "Thus, we aimed to compare knee muscle strength and movement biomechanics (walking/forward lunge) between individuals with (symptomatic) and without (asymptomatic) knee pain at least three years after ACL reconstruction. In addition, we assessed self-reported knee function, current knee pain during testing, activity level and pressure pain sensitivity to describe group differences in these parameters. We hypothesized that symptomatic individuals with ACL reconstruction have weaker quadriceps muscles and develop lower muscle forces and knee joint loading during walking and forward lunge compared to their asymptomatic counterparts."
**Methods**				
Study design	4	Present key elements of study design early in the paper	4	Lines 79-81: "This cross-sectional study, conducted at The Parker Institute, Bispebjerg and Frederiksberg Hospital, Copenhagen, Denmark, adhered to the Declaration of Helsinki and received approval from the health research ethics committee of the Capital Region of Denmark (H-20060332)."

Setting	5	Describe the setting, locations, and relevant dates, including periods of recruitment, exposure, follow-up, and data collection	4-5	Lines 79-81: "This cross-sectional study, conducted at ..."<br><br>Lines 87-92: "*Participants*<br>We identified participants who underwent ACL reconstruction at designated hospitals from 2013 to 2019 via the Danish Ligament Reconstruction Register[36]. This timeframe was chosen to balance the need for an adequate sample with minimizing the inclusion of participants with long time since surgery. Invitations were dispatched electronically. The recruitment period ran from June 2021 and June 2022."
Participants	6	(*a*) Give the eligibility criteria, and the sources and methods of selection of participants	5	Lines 92-105: "Interested respondents underwent a telephone pre-screening before receiving an invitation to a clinical screening examination for inclusion purposes. Eligible participants were aged 18 to 40 years at the time of primary ACL reconstruction (semitendinosus-gracilis tendon graft, single-bundle), with a post-surgery time of at least 3 years and a current body mass index (BMI) of ≤30. Symptomatic participants were defined by an average knee pain score of at least 3 on a 0-10 verbal rating scale (VRS) in the reconstructed knee during activities of daily living (ADL) in the past week, while asymptomatic participants were defined by a pain score of 0. Pain levels of 1 or 2 were considered minimal and unlikely to interfere with daily life, based on clinical experience, and were excluded to enhance the distinction between symptomatic and asymptomatic groups. Major exclusion criteria included known neuromuscular diseases, severe cartilage lesions at the time of injury, surgery on the contralateral knee, and musculoskeletal pain beyond the injured knee. All participants provided written informed consent prior to participating in any study-related procedures."
Variables	7	Clearly define all outcomes, exposures, predictors, potential confounders, and effect modifiers. Give diagnostic criteria, if applicable	6	Lines 126-131: "All outcomes were obtained from the participants' ACL reconstructed knee. The primary outcome was maximal isometric quadriceps muscle strength. Key secondary outcomes were peak extensor moment, peak quadriceps muscle force, and peak total knee joint contact force, assessed during walking and forward lunge."

				All additional outcomes were classified as other secondary outcomes and included: 1) The maximal isometric hamstring muscle strength, 2) the five Knee Injury and Osteoarthritis Outcome Score (KOOS), 3) the International Knee Documentation Committee (IKDC), 4) the intermittent and constant osteoarthritis pain (ICOAP) total score and two subscales, 5) the change in Tegner scores from the pre-injury activity level to the current activity level, 6) the pressure pain detection threshold (PDT) and pressure pain tolerance threshold (PTT) defined as mean of the three measurements, 7) the peak knee flexion angle during walking and forward lunge, 8) the walking speed, 9) the forward lunge foot-ground contact time, 10) Current knee pain (VRS 0-10) on the reconstructed side during muscle strength testing and walking/forward lunge.
Data sources/ measurement	8*	For each variable of interest, give sources of data and details of methods of assessment (measurement). Describe comparability of assessment methods if there is more than one group	6-10	All measurements and data collection were performed identically for the two groups. Details on the methods are described in the manuscript section *Methods* and the electronic *Supplementary material*.
Bias	9	Describe any efforts to address potential sources of bias	4+10	The study protocol was made publicly available on The Parker Institute's website (https://www.parkerinst.dk/sites/default/files/study_protocol_appi2-pt-2020-02_ver_1.2_06122021.pdf) prior to participant inclusion. All data were stored in an electronic database. A statistical analysis plan was written and publicly available online (https://www.parkerinst.dk/sites/default/files/sap_appi2-pt-2020-02_mirakos_ver_1.0.pdf) before conducting any data analyses. All questionnaires were filled out by the participants privately and directly into the electronic database (hidden from the experimental testers). All experimental tests were done in a standardized manner with identical verbal instructions. Allocation to the two study groups was done by the clinician at the clinical examination. Testers responsible for the experimental measurements described below were not explicitly informed about which group the participants belonged to. However, total blinding was impossible as the

				participants were asked about their current knee pain levels during the experiments.
Study size	10	Explain how the study size was arrived at	5-6	The sample size estimation was carefully described in the study protocol, statistical analysis plan, and in the manuscript. It was based on pragmatic considerations and due to the low prevalence of symptomatic participants, the sample size was re-calculated midwise the recruitment/data collection period. We adjusted the allocation ratio from 1:1 to 1:3.
Quantitative variables	11	Explain how quantitative variables were handled in the analyses. If applicable, describe which groupings were chosen and why	4+10<br>7-8	All variables and analyses were defined and described prior and made publicly available (https://www.parkerinst.dk/sites/default/files/study_protocol_appi2-pt-2020-02_ver_1.2_06122021.pdf and https://www.parkerinst.dk/sites/default/files/sap_appi2-pt-2020-02_mirakos_ver_1.0.pdf). In addition to our primary outcome (maximal isometric quadriceps muscle strength), we had 6 key secondary movement biomechanics outcomes, which were extracted from well-defined movement events detected in the curves of the knee joint moment, quadriceps force, and total knee contact force. These parameters were averaged across the selected trials for each participant (i.e. 6 walking trials and 3 forward lunge trials – see *Methods*, page 7-8). The current knee pain assessed during testing was also calculated as an average of the pain scores reported during the trials included in the analysis (see, page 8).
Statistical methods	12	(*a*) Describe all statistical methods, including those used to control for confounding	10-11<br>+<br>15	Lines 230-233: “The analysis was performed according to the SAP (online supplemental material) that was publicly available online (http://www.parkerinst.dk/ongoing-projects/statistical-analysis-plan-mirakos) before conducting any data analyses.”<br><br>The primary analysis applied for the estimation of between-group (symptomatic versus asymptomatic) differences of all selected outcomes was an analysis of covariance (ANCOVA) for continuous data. The results are reported as mean ± standard deviation (SD), mean differences with 95% confidence interval (CI). Categorical data and counts (percentages) were analyzed using Chi-square statistics comparing distributions between groups. The level of significance

				was set to 0.05. The analyses were done using the statistical software SAS version 9.4 (SAS Institute Inc., Cary, NC, USA)."<br><br>As stated in our statistical analysis plan, no adjustments for confounding covariates were applied in our statistical analyses. This was also stated in our Discussion, page 15: "Moreover, while no adjustments for confounding covariates were made in our analyses, we acknowledge the potential influence of measured and unmeasured variables on our results. However, we have not identified any measured covariates as potential confounders."
		(*b*) Describe any methods used to examine subgroups and interactions		N/A
		(*c*) Explain how missing data were addressed		Potential risks of missing data and how to handle that were addressed in our statistical analysis plan. However, no data were missing.
		(*d*) If applicable, describe analytical methods taking account of sampling strategy		N/A
		(*e*) Describe any sensitivity analyses		N/A
**Results**				
Participants	13*	(a) Report numbers of individuals at each stage of study—eg numbers potentially eligible, examined for eligibility, confirmed eligible, included in the study, completing follow-up, and analysed		Figure 1.
		(b) Give reasons for non-participation at each stage	11	Results, Lines 245-251: "Participants and demographics<br><br>738 invitations were sent to potential participants identified in the Danish Ligament Reconstruction Register of which 244 (33%) responded and underwent telephone pre-screening (Fig. 1). After clinical screening, 122 eligible participants (30% females) were included, of which 33 were identified as symptomatic and 89 as asymptomatic. During the telephone pre-screening, five potential participants reported knee pain scores of 1 or 2 in their

				reconstructed knee during ADL in the past week. These five subjects were found ineligible due to other exclusion criteria." + Figure 1.
		(c) Consider use of a flow diagram		Figure 1
Descriptive data	14*	(a) Give characteristics of study participants (eg demographic, clinical, social) and information on exposures and potential confounders		Table 1
		(b) Indicate number of participants with missing data for each variable of interest		N/A
Outcome data	15*	Report numbers of outcome events or summary measures		Table 2 and 3
Main results	16	(*a*) Give unadjusted estimates and, if applicable, confounder-adjusted estimates and their precision (eg, 95% confidence interval). Make clear which confounders were adjusted for and why they were included		Table 2 and 3. No adjustments for confounders were done as no measured confounders were identified that potentially could impact both knee pain presence and musculoskeletal function.
		(*b*) Report category boundaries when continuous variables were categorized		N/A
		(*c*) If relevant, consider translating estimates of relative risk into absolute risk for a meaningful time period		N/A
Other analyses	17	Report other analyses done—eg analyses of subgroups and interactions, and sensitivity analyses		N/A
**Discussion**				
Key results	18	Summarise key results with reference to study objectives	12	Lines 283-288
Limitations	19	Discuss limitations of the study, taking into account sources of potential bias or imprecision. Discuss both direction and magnitude of any potential bias	15	Lines 342-362
Interpretation	20	Give a cautious overall interpretation of results considering objectives, limitations, multiplicity of	13-15	Lines 289-341

		analyses, results from similar studies, and other relevant evidence		
Generalisability	21	Discuss the generalisability (external validity) of the study results	15	Lines 362-365
**Other information**				
Funding	22	Give the source of funding and the role of the funders for the present study and, if applicable, for the original study on which the present article is based		Upon submission, the journal received the following funding statement in a separate document to ensure anonymization for peer review. This statement will be published in full upon acceptance of the manuscript.<br>"Funding: Innovation Fund Denmark (9088-00006B under the frame of ERA PerMed). Research Council of Finland (#332915) and Finnish Cultural Foundation (North Savo Regional Fund) to LS. Finnish Cultural Foundation (North Savo Regional Fund), Maire Lisko Foundation, and Instrumentarium Science Foundation to WB. The Parker Institute, Bispebjerg and Frederiksberg Hospital is supported by a core grant from the Oak Foundation (OCAY-18-774-OFIL)."

*Give information separately for exposed and unexposed groups.

Note: An Explanation and Elaboration article discusses each checklist item and gives methodological background and published examples of transparent reporting. The STROBE checklist is best used in conjunction with this article (freely available on the Web sites of PLoS Medicine at http://www.plosmedicine.org/, Annals of Internal Medicine at http://www.annals.org/, and Epidemiology at http://www.epidem.com/). Information on the STROBE Initiative is available at www.strobe-statement.org.
