# Supplementary material for: Knee muscle strength and movement biomechanics in individuals with and without knee pain after anterior cruciate ligament reconstruction: A cross‐sectional study
Source: Knee Surg Sports Traumatol Arthrosc. 2025 Feb 20;33(12):4136–47. doi: 10.1002/ksa.12630 (PMC12684341; doi:10.1002/ksa.12630)
Supplement: Supplementary file 2 — Supporting information. [file KSA-33-4136-s001.pdf]

Supplementary material

Musculoskeletal function in anterior cruciate ligament reconstructed individuals with and without knee pain: A cross-sectional study

Table of contents

Study protocol 2

Statistical analysis plan 20

Details of musculoskeletal modeling and simulation 42

Study protocol

INVESTIGATION PLAN: CROSS-SECTIONAL STUDY

MUSCULOSKELETAL FUNCTION IN ANTERIOR CRUCIATE LIGAMENT RECONSTRUCTED INDIVIDUALS WITH AND WITHOUT KNEE PAIN

**Study Director, Principal Investigator:**	Tine Alkjær, MSc, PhD[1,2]
**Investigator:**	Marius Henriksen, PT, PhD[2]
**Investigator:**	Henning Bliddal, MD, DMSc[2]
**Investigator:**	Per Hölmich, MD, DMSc[3]
**Investigator:**	Lauri Stenroth, MSc, PhD[1,2]
**Investigator:**	Henrik Aagaard, MD, PhD[4]

Sponsor:

The Parker Institute, Bispebjerg-Frederiksberg Hospital, Copenhagen, Denmark.

Affiliations:

[1]Department of Biomedical Sciences, University of Copenhagen, Copenhagen, Denmark.

[2]The Parker Institute, Bispebjerg-Frederiksberg Hospital, Copenhagen, Denmark.

[3]Sports Orthopedic Research Center – Copenhagen (SORC-C), Department of Orthopedic Surgery, Copenhagen University Hospital, Amager-Hvidovre, Denmark.

[4]Ortopaedic Department, Sealand University Hospital Koege, Koege, Denmark.

Version: 1.2

Date: 06 December 2021

Protocol revision history:

Version #	Issue date	Amendment
1.0	14.12.2020	Letter of invitation – added (section 11.6)
1.1	21.01.2021	Health research ethics committee number added (section 1.2). Information about responsibility for study initiation added (section 1.4). Specification regarding compliance of Data Protection Act added (section 9.1). Specification regarding financial issues (section 9.3).
1.2	17.05.2021	Added questionnaire "Intermittent and Constant Osteoarthritis Pain" (ICOAP) (section 7.2.4)
1.2	04.11.2021	Sample size recalculation. Expected group sizes was changed from 1:1 to 1:3 due to low prevalence of symptomatic participants. As per November 2021 we had recruited 17-54 (symptomatic-asymptomatic, respectively) (section 8.1)
1.2	06.12.2021	Per December 2021 no further participant recruitments from Amager-Hvidovre Hospital were possible. A new hospital is added to the protocol to increase the recruitment basis (section 5.3). The orthopedic surgeon at this hospital is added as investigator.

1.0 STUDY IDENTIFIER

1.1 FULL TITLE

Musculoskeletal function in anterior cruciate ligament reconstructed individuals with and without knee pain.

1.2 HEALTH RESEARCH ETHICS COMMITTEE NUMBER

H-20060332

1.3 INTERNAL PROTOCOL NUMBER

APPI2-PT-2020-02

1.4 STUDY INITIATION

Tine Alkjær, MSc, PhD, associate professor, Department of Biomedical Sciences, University of Copenhagen, Copenhagen, Denmark.

2.0 PROTOCOL SYNOPSIS

Study title	Musculoskeletal function in anterior cruciate ligament reconstructed individuals with and without knee pain.
Funder	INNOVATION FUND DENMARK under the frame of ERA PerMed: ERAPERMED2019-331 – DEEPMECHANOKNEE.
Study objectives	The objective is to compare the musculoskeletal function between ACL reconstructed individuals with ("Symptomatic") and without knee pain ("Asymptomatic").<br>The hypotheses are:<br>1) ACL reconstructed individuals without knee pain have stronger quadriceps muscles compared to those with knee pain.<br>2) ACL reconstructed individuals without knee pain develop higher quadriceps muscle forces and knee joint compressive forces during walking and forward lunging compared to those with knee pain.
Study design	Observational cross-sectional study.
Subject populations	Individuals with ACL reconstruction.
Inclusion/exclusion criteria	Inclusion criteria:<br>- Age between 18 and 40 years at the time of ACL reconstruction.<br>- Primary ACL reconstruction (semitendinosus-gracilis tendon graft).<br>- Post-surgery time of at least 3 years.<br>- A body mass index (BMI) of ≤30.<br>- Pain score of 0[Defines "Asymptomatic"] *or* at least 3 [Defines "Symptomatic"] (verbal rating scale (VRS) 0-10) in the reconstructed knee during activities of daily living (ADL) within the last week.<br>Exclusion criteria:<br>- Known neuromuscular diseases.<br>- Cartilage lesions ICRS grade 4 (full thickness).<br>- ACL reconstruction or other major surgery to the other knee<br>- Congenital deformities in the lower extremities preventing full participation in the tests.<br>- Musculoskeletal pain in the lower extremity other than the injured knee.<br>- Any other condition that in the opinion of the investigator makes a potential participant unfit for participation or conditions that puts a potential participant at risk by participation.
Observation	Individuals with an ACL reconstruction in one knee with and without knee pain will be identified and invited to participate in the study where their maximal isometric quadriceps and hamstring muscle strength will be assessed (Biodex System4 Pro, Biodex Medical System, NY, USA). Walking/forward lunge biomechanics will be assessed using standard three dimensional movement analyses (Vicon MX, Vicon Motion Systems, Oxford, UK) and knee joint compression force and quadriceps muscle force production during movement will be estimated from further biomechanical modelling. Knee pain during walking is assessed by a 100 mm visual analogue scale. Pressure pain sensitivity is assessed using computerised cuff pressure algometry. Structural signs of knee OA are determined from standing standardised x-rays of the knees using the Kellgren-Lawrence grading system. Self-reported knee function is obtained by questionnaires

	(IKDC, KOOS) pain and activity level is assessed using the ICOAP and the Tegner score. This study is observational and no interventions will be applied.
Outcomes	Primary outcome: Maximal isometric quadriceps muscle strength.<br>Secondary outcomes: Knee joint biomechanics during walking/forward lunging. Maximal isometric hamstring muscle strength. Knee pain. Pressure pain sensitivity. Self-reported knee function and activity level. Radiographic knee OA level.
Sample size	To detect a group difference of 0.3 Nm/kg in the primary outcome with a common standard deviation of 0.5 Nm/kg, a sample size of 120 with a 1:3 group allocation (n=30 symptomatic; n=90 asymptomatic) will reach a power of 80.6%. Thus, a total sample size of n=120 (30/90 symptomatic/asymptomatic) will be applied.
Study duration	Time for preparation of the study (months): 2<br>Recruitment period (months): 4<br>First participant tested to last participant tested (months): 24<br>Time for data clearance and analysis (months): 9<br>Duration of the entire study (months): 36
Safety evaluation	No safety issues.
Statistical analysis	All outcomes: Comparison between groups: with and without pain (ANCOVA).
Data and safety monitoring plan	No safety issues.<br>The data management plan will comply with the common rules regarding data protection (General Data Protection Regulation (GDPR)). The study will be conducted in accordance with Danish law, the Helsinki declaration, and local research ethics committee requirements.
Participating centres	To be involved (n): 1, in Denmark.

3.0 INTRODUCTION

3.1 BACKGROUND AND RATIONALE

Knee osteoarthritis (OA) is the most common joint disease and a significant contributor to global disability [1]. The known knee OA risk factors include obesity, surgery, occupational load and injury [2,3]. Anterior cruciate ligament (ACL) rupture is a common knee injury [4,5] and the incidence is increasing, particularly among young people [6]. ACL injury affects the knee joint function and increases the risk of knee OA development [7-10] even at a young age, which prolongs the period of impaired function and pain [11]. Most research has focused on radiographic knee OA while fewer studies have investigated the prevalence of symptomatic knee OA after ACL injury [12]. It is important to discriminate between radiographic and symptomatic knee OA, as knee pain is a decisive criterion to diagnose knee OA [13], whereas radiographic changes more serve as a confirmatory measure. Indeed, the Framingham study showed that the prevalence of radiographic changes (indicative of OA) in the population older than 63 years was 33% whereas the prevalence of symptoms was only 9% [14]. A recent MRI study of 230 asymptomatic knees reported that 97% of these showed abnormalities in at least one knee structure [15]. This emphasizes that image-based signs of knee OA are not always accompanied by pain and OA symptoms.

Conventionally, mechanical joint loading is proposed as a key mechanism contributing to development and progression of OA [16,17]. Thus, the knee joint loading during dynamic tasks in the ACL injured population has been studied extensively due to the supposed link between the knee joint compressive forces and onset of post traumatic knee OA [18-23]. However, the evidence for a causal link between knee joint loading and knee OA development and progression is weak [24,25]. Furthermore, a 15-year follow-up study, showed that ACL reconstructed persons returning to pivoting sport (presumably associated with high loads) had reduced odds of developing knee OA and had better self-reported function in activities of daily living [26]. On the other hand, data suggest that ACL reconstructed individuals develop different adaptive neuromuscular functions [27,28], and it is possible that other mechanical factors than loading magnitude are implicated in the development of knee OA. Such other biomechanical factors may include force dissipation capacity of the musculoskeletal system [29], micro-incoordination [30], muscle strength and other aspects of muscle function. Low quadriceps muscle strength is associated with an increased risk of symptomatic and functional impairment in people with and at risk of radiographic knee OA [31]. The quadriceps muscle strength and function is impaired after ACL injury and strength deficits persist even after ligament reconstruction [32-34]. Altogether, there are indices and a common agreement that poor musculoskeletal function is associated with increased risk of development of both symptomatic and radiographic knee OA, and that an ACL injury and reconstruction may lead to unfavorable changes in the musculoskeletal function accelerating the development of symptoms and/or degenerative OA changes. One study has compared individuals with definitive radiographic OA with and without symptoms and found that the symptomatic group had lower muscle strength and walking biomechanics indicative of a "stiffer" gait, possibly reflecting protective neuromuscular adaptations in the walking pattern [35]. As ACL injuries increase the risk of OA (symptomatic and radiographic) later in life, the musculoskeletal function may be changed alongside with the early onset of symptoms but before definitive radiographic OA is present. Thus, the present study will compare the musculoskeletal function between ACL reconstructed individuals with and without knee pain. By this we can deepen our understanding of the role of the musculoskeletal function in relation to development and progression of knee OA.

4.0 STUDY OBJECTIVE, HYPOTHESES AND OUTCOMES

The objective of the present study is to compare the musculoskeletal function between ACL reconstructed individuals with and without knee pain.

Musculoskeletal function will be assessed by

- Muscle strength of the knee extensor muscle (quadriceps)
- Biomechanics of the knee and quadriceps muscle during level walking and a forward lunge movement

The hypotheses are:

1) ACL reconstructed individuals without knee pain have stronger quadriceps muscles compared to those with knee pain.
2) ACL reconstructed individuals without knee pain develop higher quadriceps muscle forces and knee joint compressive forces during walking and forward lunging compared to those with knee pain.

The rationale for the hypotheses is based on research documenting that quadriceps muscle weakness is associated with an increased risk of symptomatic and functional impairment in people with and at risk for radiographic knee OA[31,35], and that knee joint pain has a negative impact on quadriceps muscle activation and force production[36].

4.1 PRIMARY AND SECONDARY OUTCOMES

Primary outcome:

- Maximal isometric quadriceps muscle strength.

Secondary outcomes:

- Knee joint biomechanics during walking/forward lunging.
- Knee flexor (hamstring) muscle strength.
- Knee pain.
- Pressure pain sensitivity.
- Self-reported knee function and activity level.
- Radiographic knee OA level.

5.0 STUDY DESIGN

5.1 DESCRIPTION OF THE PROTOCOL

This is an observational cross-sectional study investigating the musculoskeletal function in two groups of ACL reconstructed persons discriminated by the presence of knee pain. The participants are invited for one study visit at which all data are collected.

5.2 PARTICIPANTS

In total, 100 (see sample size calculation in section 8.1) ACL reconstructed persons will be identified from the Danish Ligament Reconstruction Register and invited to participate in the study.

As we aim to compare participants with and without knee pain the eligibility criteria are as follows:

Participants with knee pain ("Symptomatic group"):

Inclusion criteria:

- Age between 18 and 40 years at the time of ACL reconstruction.
- Primary ACL reconstruction using the semitendinosus-gracilis tendon graft.
- Post-surgery time of at least 3 years.
- Current body mass index (BMI) of ≤30.
- Pain score of at least 3 (verbal rating scale (VRS) 0-10) in the reconstructed knee during activities of daily living (ADL) within the last week.

Participants without knee pain ("Asymptomatic group"):

Inclusion criteria:

- Age between 18 and 40 years at the time of ACL reconstruction.
- Primary ACL reconstruction using the semitendinosus-gracilis tendon graft.
- Post-surgery time of at least 3 years.
- Current body mass index (BMI) of ≤30.
- Pain score of 0 (VRS 0-10) in the reconstructed knee during activities of daily living (ADL) within the last week.

For both groups, the exclusion criteria are the same:

Exclusion criteria:

- Known neuromuscular diseases.
- Evidence of cartilage lesions ICRS grade 4 (full thickness) from MRI at time of ACL reconstruction or documented peri-surgically.
- ACL reconstruction or other major surgery to the other knee.
- Congenital deformities in the lower extremities preventing full participation in the tests.
- Current musculoskeletal pain in other regions of the lower extremity other than the injured knee.
- Any other condition that in the opinion of the investigator makes a potential participant unfit for participation or conditions that puts a potential participant at risk by participation.

All participants will receive written (appended) and oral information about the purpose of the study, the study protocol, the duration and the expectations. They will be offered time to consider participation and asked to sign an informed consent form (appended) before any study related procedures are done.

5.3 RECRUITMENT

The participants will be recruited from the Danish Ligament Reconstruction Register, starting with individuals who have had reconstruction surgery at the Department of Orthopedic Surgery, Copenhagen University Hospital, Amager-Hvidovre, Copenhagen, Denmark and Ortopaedic Department, Sealand University Hospital Koege, Koege, Denmark. If recruitment of the scheduled number of participants cannot be reached within a reasonable timeframe, individuals treated at other hospitals in Denmark may be necessary to identify and contact via the Danish Ligament Reconstruction Register.

Also, advertisements in local newspapers, on the participating department's webpages, and on social media may be used if recruitment direct from the Danish Ligament Reconstruction Register is insufficient or too slow (appended).

5.4 PRE-SCREENING AND SCREENING PROCEDURES

Potential participants are contacted and pre-screened and screened as follows:

1) Letter of invitation send via digital mail (e-Boks) stating the main criteria for participation.
2) Potential participants contact the research team in case they are interested.
3) Potential participants are invited for a clinical screening examination at Bispebjerg-Frederiksberg Hospital/The Parker Institute, for the purpose of inclusion (see section 6.0 regarding study procedures).
4) Eligible participants are invited to an X-ray examination of both knees and an examination of musculoskeletal function (see section 7.0 regarding measurements).

6.0 STUDY PROCEDURES

6.1 ORAL INFORMATION

The oral information visit will be organised as an individual session with an investigator (or his/her delegate) at the OA outpatient clinic at The Parker Institute. Potential participants have the right to bring next of kin or another person of the participant's choice with him/her to the oral information visit.

The information will include that

- Participation in the study is voluntary
- Participants have the right to minimum 24 hours reflection time before deciding to either sign the informed consent or decline
- Participants can, at any time and without giving any reason, withdraw from the study without affecting the potential participant's right to current or future treatment

Further, the oral information will include: aim, procedures, potential benefits and risks when participating in the study, procedures for random findings during the project, procedures for securing the participants privacy and data protection, information on the study organisation, funding, as well as contact information on the primary investigator and other key investigators.

The investigator will make sure that participants have received and understood the information given to them. Furthermore, the investigator will make sure they are aware that they have the right to minimum 24 hours reflection time before signing the informed consent.

The written information material will be provided.

6.2 SCREENING VISIT

At the screening visit, the participants provide written informed consent and undergo the screening procedures. The screening procedures will only be done upon signed informed consent.

At the screening visit, the following procedures will be done in this order:

1. Provision of signed informed consent
2. Assessment of in- and exclusion criteria, including
    a. Measurement of height and body mass

b. Clinical examination by an investigator
c. Interview about medical history

Participants who meet all inclusion criteria and who do not have exclusions will be scheduled for a measurement visit.

6.3 MEASUREMENT VISIT

At the measurement visit, the following procedures will be completed (see section 7.0 for detailed descriptions):

- Knee radiographs (section 7.1)
- Questionnaires (section 7.2)
- Muscle strength test (section 7.3)
- Walking and forward lunge biomechanics test (section 7.4)
- Pain sensitivity (section 7.5)

All measurements will be performed at The Parker Institute/Bispebjerg-Frederiksberg Hospital, Copenhagen, Denmark.

7.0 OUTCOME MEASUREMENTS

7.1 KNEE RADIOGRAPHS

To assess the radiographic level of knee OA bilateral standing knee radiographs will be acquired. The radiographic recordings will be done at Frederiksberg Hospital. The evaluation of radiographic signs of knee OA are done according to Kellgren-Lawrence grading [37].

7.2 QUESTIONNAIRES

Information about the participants' perceived knee function and level of activity will be assessed by questionnaires developed for evaluation of ACL injury and knee OA: The International Knee Documentation Committee (IKDC) [38] and the Knee Injury and Osteoarthritis Outcome Scale (KOOS) [39], the Intermittent and Constant Osteoarthritis Pain questionnaire (ICOAP)[40], and the Tegner score [41] will be filled out by the participants at the study visit. All questionnaires are attached to this protocol.

7.2.1 IKDC

The IKDC questionnaire is an instrument to assess patients with a variety of knee disorders including ligamentous and meniscal injuries as well as patellofemoral pain and osteoarthritis[38]. The questionnaire consists of three subscales: symptoms (7 items), sports activity (2 items), and knee function (2 items) and provides an overall function score. The scores are obtained by summing the individual items and then convert the crude total to a scaled number that ranges from 0 to 100. This final number represents a measure of function with higher scores representing higher levels of function. Thus, a score of 100 reflects no functional limitations.

7.2.2 KOOS

The Knee injury and Osteoarthritis Outcome Score (KOOS), a disease-specific instrument, is an extension of the Western Ontario and McMaster Universities Osteoarthritis Index (WOMAC) and designed to assess health related quality of life (QoL) in patients with knee injuries and knee OA[39]. The KOOS consists of 42 items covering five domains, namely, Pain (9 items), Symptoms (7 items), Activities of Daily Living (ADL) (17 items), Sports and Recreation (5 items), and knee-

related QoL (4 items). The KOOS adopts a five-point Likert scale scoring system (ranging from 0 (least severe) to 4 (most severe)).

A normalized score is calculated for each domain with 100 indicating no symptoms and functional impairment and 0 indicating extreme symptoms and functional impairment. In accordance with the user guide (http://www.koos.nu), if the number of missing items is less than or equal to 2 in a subscale they will be substituted by the average item value for that subscale. If more than two items of the subscale are omitted the response will be considered invalid and no subscale score calculated.

7.2.3 Tegner score

The Tegner activity scale is an instrument to measure activity following knee injuries[41]. It grades activity based on work and sports activities on a scale of 0 to 10 one-item score. Zero represents disability due to knee problems and 10 represents competitive sports (soccer - national and international elite level). The subjects report the level of participation that best describes their current level of activity and that before injury.

7.2.4 ICOAP

The Intermittent and Constant Osteoarthritis Pain questionnaire (ICOAP) is a diagnosis-specific 11-item questionnaire designed to assess the pain experience within the last week among people suffering from knee and hip OA[40]. The questionnaire is divided into two domains, a 5-item scale for constant pain and a 6-item scale for intermittent pain (so-called ‘‘pain that comes and goes’’). Each domain captures pain intensity as well as related distress and the impact of OA pain on quality of life. For each of these pain types, single items assess pain intensity, effect on sleep, impact on quality of life, extent to which the pain ‘frustrates or annoys’, and the extent to which it ‘worries or upsets’. For pain that comes and goes, two additional items ask respondents to report the frequency of pain and the degree to which the pain could be predicted. All items are scored on anchored rating scales with five levels of response (0–4) – for questions asking about intensity, response options are ‘not at all’ (0), to ‘extremely’ (4), while those that asked about frequency has the following response options: ‘never’ (0), to ‘very often’ (4). A score is separately produced for the constant pain subscale (0–20) and the intermittent pain subscale (0–24), and for total pain (0–44). Normalized scores for the two subscales and for the total pain score, from 0 (no pain) to 100 (extreme pain), are calculated.

7.3 MUSCLE STRENGTH TEST

Isometric quadriceps and hamstring muscle strength will be assessed using an isokinetic dynamometer (Biodex System4 Pro, Biodex Medical System, NY, USA). The dynamometer records the torque (Nm) produced by isometric muscle contractions. The participants are seated in a rigid chair firmly strapped to the seat at the hip and distal thigh. The rotation axis of the dynamometer is visually aligned to the lateral femoral epicondyle and the lower leg attached to the lever arm of the dynamometer. The lever arm is placed just above the lateral malleolus and fixed with a cuff. Prior to testing, 15 min. of warm-up will be applied to familiarize the subjects to the dynamometer and the test procedures. Maximal voluntary isometric contractions (MVICs) of the quadriceps and hamstrings, respectively, will be done at 60° knee flexion. The participants are asked to perform the MVICs with maximal effort and verbal feedback and encouragement will be provided during testing that comprises three repetitions of which the highest peak torque value will be defined as the maximal quadriceps/hamstring muscle strength and reported as body mass normalized values (Nm/kg)[42].

7.4 WALKING AND FORWARD LUNGE BIOMECHANICS TEST

All participants will have their walking and forward lunge movement pattern assessed using standard three-dimensional motion capture and software (Vicon MX, Vicon Motion Systems, Oxford, UK). Small reflective markers are attached to the participants' skin over well-defined anatomical landmarks and then the movements (walking/forward lunge) are performed.

Walking and forward lunge kinematics and kinetics of the ankle, knee and hip joints of both legs will be quantified using a standard inverse dynamics calculation model (Vicon Nexus ver 2.10, Vicon Motion Systems, Oxford, UK). Further computational musculoskeletal modeling of the biomechanical data will be used to estimate the knee compression force and the quadriceps muscle force developed during the stance/contact phase of walking/forward lunge.

7.4.1 Walking biomechanics

During walking the participants walk across two ground reaction for plates (AMTI OR 6-5-1000, Watertown, MA, USA) mounted in a 10 m long walkway at a self-selected speed (target speed). The target speed will be determined during the trial habituation procedure. The walking speed are recorded during each trial that are repeated until a sufficient amount of acceptable trials (walking speed within ±0.1 km/h of the target speed) are captured and stored for further processing.

7.4.2 Forward lunge biomechanics

The participants will be instructed to perform forward lunge movements at maximal pace ("as fast as possible"). The forward lunge movements are performed by taking one step forward, placing the foot on the force plate, flexing the knee to 90° and subsequently push backwards into the starting position, while having hands on the back of the head, the upper body perpendicular to the ground, and the opposite foot maintaining ground contact. Three forward lunge movements will be recorded with a short resting period (~60 s) in between.

7.4.3 Knee pain during movement

The current knee pain during walking/forward lunging will be assessed by a VRS 0-10 immediately after each walking/lunging trial.

7.5 PAIN SENSITIVITY

The pain sensitivity will be assessed by computerised cuff pressure algometry (CPA)[43]. A double-chambered Tourniquet cuff is wrapped around the calf by the gastrocnemius muscles of the lower extremity of the ACL reconstructed leg. A computer controlled compressor inflates the cuff with air at 1 kPa/s until the person reports the first sensation of pain by pressing a push-button[44]. The recorded pressure defines the pressure pain threshold (PPT) measured in kPa.

8.0 STATISTICAL CONSIDERATIONS

8.1 SAMPLE SIZE

We will compare the quadriceps muscle strength between two groups of ACL reconstructed individuals: 1) with and 2) without knee pain.

The variance in this population is unknown, while our sample size estimation will be pragmatic. To detect a group difference of 0.3 Nm/kg in the primary outcome with a common standard deviation of 0.5 Nm/kg, a sample size of 120 with a 1:3 group allocation (n=30 symptomatic; n=90

asymptomatic) will reach a power of 80.6%. Thus, a total sample size of n=120 (30/90) will be applied.

8.2 GROUP COMPARISONS

All comparisons between groups (with and without knee pain) will be analysed using an analysis of covariance (ANCOVA). The results will be reported as mean ± SD, mean differences with 95% confidence interval (CI) and the level of significance is set to 0.05.

9.0 REGULATORY STANDARDS AND DATA MANAGEMENT

9.1 NOTIFICATION TO THE DANISH DATA PROTECTION AGENCY

This study will follow the common rules regarding data protection i.e. the General Data Protection Regulation (GDPR) and be conducted in accordance with Danish law, the Helsinki declaration, and local research ethics committee requirements. Thus, the processing of personal data is carried out in compliance with Regulation No 2016/679 of the European Parliament and of the Council of 27 April 2016 on the protection of natural persons with regard to the processing of personal data and on the free movement of such data, the Data Protection Act (in Danish: “databeskyttelsesloven”) and the Danish Health Care Act (in Danish: “sundhedsloven”). This process will ensure that the data management of the study comply with the data protection regulation.

Participant medical information obtained by this study is confidential, and disclosure to third parties other than those noted below is prohibited.

With the participant’s permission, information may be shared with his or her personal physician or with other medical personnel responsible for the participant’s welfare.

Publication of data from this study will not include names, recognizable photos, personal information or other data that compromises the anonymity of participating participants.

9.2 QUALITY ASSURANCE

All data will be entered into a study database for analysis and reporting. Any data captured electronically will be stored electronically in a separate database according to standard procedures at secured servers. Upon completion of data entry, the databases will be checked to ensure acceptable accuracy and completeness.

Individuals involved in study evaluations will be trained to perform the efficacy evaluations and activity measurements described in the protocol.

9.3 FINANCING AND INSURANCE INFORMATION

This study is a part of a project entitled “A novel tool for personalised and socio-economically optimal treatment planning for patients with osteoarthritis” that received a grant from ERA PerMed: ERAPERMED2019-331 – DEEPMECHANOKNEE. Tine Alkjær, associate professor, Department of Biomedical Sciences, University of Copenhagen, is the principal investigator of the work package (WP) “Motion analysis and musculoskeletal modeling to characterize the effect of obesity, weight loss and anterior cruciate ligament injury on the onset and progression of osteoarthritis”. The national funding agency (Innovation Fund Denmark) has granted the Danish WP DKK: 2.890.757. This grant is transferred to the Department of Biomedical Sciences, University of Copenhagen. This grant will primarily be used to cover salary for a post doc (Lauri Stenroth, study investigator) employed at Department of Biomedical Sciences, University of Copenhagen, Copenhagen,

Denmark and running cost. In case further funding will be granted to the study, the Health research ethics committee and the study participants will be informed.

The participants are insured by the Danish Patient Insurance Association. Financing and insurance issues are addressed in the written information material.

The research partners involved in the study has no conflicts of interest to declare.

9.4 PUBLICATION

All positive, negative and nonconclusive results will be published in relevant international scientific peer-reviewed journal and presented at national and international conferences. The study findings will be conveyed in a transparent way.

10.0 ETHICS

10.1 GENERAL CONSIDERATIONS

All potential participants are informed, both orally and in writing, about the study purpose, its process and potential risks, as well as costs and benefits of participation. All participants are informed of their rights to withdraw from the study at any time without this influencing any future investigations and/or treatments at any site or by some of the members of the study group. After the information is delivered, read and understood, the participant gives voluntary informed consent by signing a consent form before study participation can take place. The potential participants have at least 24 hours to consider participating in the study.

It is the investigators' opinion that the knowledge and potential individual benefit gained by participation in this study is commensurate with the efforts and difficulties associated with participation. Below are specific research ethics considerations related to information, consent, interventions, and outcome assessments.

10.2 STANDARD TREATMENT

There are no restrictions about medical treatment/other treatments.

10.3 ORAL INFORMATION

The oral information is based on the written information and will be given in an easily understandable language without technical or value-laden terms. The information will be given in a considerate way that is tailored to each potential study participants. The aim is that the conversation takes place without interference. It is the responsibility of the interviewer to ensure that the potential participant has understood the information. The information interview is performed by the investigator or in her absence by a designated delegate.

10.4 WRITTEN INFORMATION

A written information material has been prepared and is attached to this protocol.

10.5 INFORMED CONSENT

Consent to participation in the study is given on the basis of the written and oral information.

An informed consent form (ICF) has been prepared. The form must be signed and dated by the participants prior to participation in the study. A copy of the form is provided to the participants. The investigator or her designated delegates can receive the signed consent form.

The source documentation and case report forms (CRFs) will document for each participant that informed consent was obtained prior to participation in the study. The signed ICF must remain in each participant's study file and must be available for verification by study monitors at any time.

10.6 RESEARCH ETHICS – THE MEASUREMENTS

The measurements regarding muscle strength, biomechanics, pain sensitivity and questionnaires are non-invasive and not associated with any predictable harms or risks to the participants. When measuring pain sensitivity, a mild, short-term pain occurs, which disappears as soon as the participant senses the pain threshold is reached.

The radiographical examination of the of the participants' knee joints will give the participants a minimal extra dosis of radiation. The effective dose for a single x-ray image of both knees is approximately 3 μSv. The annual background radiation in Denmark is approximately 3000 μSv (≈ 8 μSv / day). When exposed to a dose of 1 Sv (1,000000 μSv), the risk of causing a cancerous disease increases by 5% over the average risk in the population. The risk increments following exposure in this study is 3 μSv (x-ray both knees) can be calculated as 0.000003 Sv x 5% per Sv = 0.00000015% that should be added to the lifetime risk of dying from cancer of 25% in Denmark, that theoretically will change to 25.00000015%.

All measurements are obtained according to well-known methods and are considered justifiable from a health research ethics perspective.

10.7 RESEARCH ETHICS APPROVAL

The study protocol and all attached documents will be submitted to the health research ethics committee to apply for approval.

Furthermore, we will conduct the study in accordance with Danish law, the Helsinki declaration, and local research ethics committee requirements.

11.0 APPENDICES

11.1 APPENDIX : QUESTIONNAIRES

11.2 APPENDIX : WRITTEN INFORMATION MATERIAL

11.3 APPENDIX : INFORMED CONSENT FORM

11.4 APPENDIX: RECRUITMENT MATERIAL

11.5 APPENDIX: GUIDELINES FOR ORAL INFORMATION

11.6 APPENDIX: LETTER OF INVITATION - DIGITAL MAIL (E-BOKS)

12.0 REFERENCES

1. Cross M, Smith E, Hoy D, et al. The global burden of hip and knee osteoarthritis: estimates from the global burden of disease 2010 study. *Ann Rheum Dis.* 2014;73(7):1323-1330.
2. Felson DT. Clinical practice. Osteoarthritis of the knee. *N Engl J Med.* 2006;354(8):841-848.
3. Blagojevic M, Jinks C, Jeffery A, Jordan KP. Risk factors for onset of osteoarthritis of the knee in older adults: a systematic review and meta-analysis. *Osteoarthritis Cartilage.* 2010;18(1):24-33.
4. Kaeding CC, Leger-St-Jean B, Magnussen RA. Epidemiology and Diagnosis of Anterior Cruciate Ligament Injuries. *Clin Sports Med.* 2017;36(1):1-8.
5. Moses B, Orchard J, Orchard J. Systematic review: Annual incidence of ACL injury and surgery in various populations. *Res Sports Med.* 2012;20(3-4):157-179.
6. Werner BC, Yang S, Looney AM, Gwathmey FW, Jr. Trends in Pediatric and Adolescent Anterior Cruciate Ligament Injury and Reconstruction. *J Pediatr Orthop.* 2016;36(5):447-452.
7. Lohmander LS, Englund PM, Dahl LL, Roos EM. The long-term consequence of anterior cruciate ligament and meniscus injuries: osteoarthritis. *Am J Sports Med.* 2007;35(10):1756-1769.
8. Lohmander LS, Ostenberg A, Englund M, Roos H. High prevalence of knee osteoarthritis, pain, and functional limitations in female soccer players twelve years after anterior cruciate ligament injury. *Arthritis Rheum.* 2004;50(10):3145-3152.
9. Whittaker JL, Woodhouse LJ, Nettel-Aguirre A, Emery CA. Outcomes associated with early post-traumatic osteoarthritis and other negative health consequences 3-10 years following knee joint injury in youth sport. *Osteoarthritis Cartilage.* 2015;23(7):1122-1129.
10. Riordan EA, Frobell RB, Roemer FW, Hunter DJ. The health and structural consequences of acute knee injuries involving rupture of the anterior cruciate ligament. *Rheum Dis Clin North Am.* 2013;39(1):107-122.
11. Stiebel M, Miller LE, Block JE. Post-traumatic knee osteoarthritis in the young patient: therapeutic dilemmas and emerging technologies. *Open Access J Sports Med.* 2014;5:73-79.
12. Lie MM, Risberg MA, Storheim K, Engebretsen L, Oiestad BE. What's the rate of knee osteoarthritis 10 years after anterior cruciate ligament injury? An updated systematic review. *Br J Sports Med.* 2019;53(18):1162-1167.
13. Altman R, Asch E, Bloch D, et al. Development of criteria for the classification and reporting of osteoarthritis. Classification of osteoarthritis of the knee. Diagnostic and Therapeutic Criteria Committee of the American Rheumatism Association. *Arthritis Rheum.* 1986;29(8):1039-1049.
14. Felson DT. The epidemiology of knee osteoarthritis: results from the Framingham Osteoarthritis Study. *Semin Arthritis Rheum.* 1990;20(3 Suppl 1):42-50.
15. Horga LM, Hirschmann AC, Henckel J, et al. Prevalence of abnormal findings in 230 knees of asymptomatic adults using 3.0 T MRI. *Skeletal Radiol.* 2020.
16. Felson DT. Osteoarthritis as a disease of mechanics. *Osteoarthritis Cartilage.* 2013;21(1):10-15.
17. Andriacchi TP, Mundermann A. The role of ambulatory mechanics in the initiation and progression of knee osteoarthritis. *Curr Opin Rheumatol.* 2006;18(5):514-518.
18. Wellsandt E, Khandha A, Manal K, Axe MJ, Buchanan TS, Snyder-Mackler L. Predictors of knee joint loading after anterior cruciate ligament reconstruction. *J Orthop Res.* 2017;35(3):651-656.

19. Tsai LC, McLean S, Colletti PM, Powers CM. Greater muscle co-contraction results in increased tibiofemoral compressive forces in females who have undergone anterior cruciate ligament reconstruction. *J Orthop Res.* 2012;30(12):2007-2014.
20. Capin JJ, Khandha A, Zarzycki R, et al. Gait mechanics and tibiofemoral loading in men of the ACL-SPORTS randomized control trial. *J Orthop Res.* 2018;36(9):2364-2372.
21. Capin JJ, Khandha A, Zarzycki R, Manal K, Buchanan TS, Snyder-Mackler L. Gait Mechanics After ACL Reconstruction Differ According to Medial Meniscal Treatment. *J Bone Joint Surg Am.* 2018;100(14):1209-1216.
22. Gardinier ES, Manal K, Buchanan TS, Snyder-Mackler L. Altered loading in the injured knee after ACL rupture. *J Orthop Res.* 2013;31(3):458-464.
23. Slater LV, Hart JM, Kelly AR, Kuenze CM. Progressive Changes in Walking Kinematics and Kinetics After Anterior Cruciate Ligament Injury and Reconstruction: A Review and Meta-Analysis. *J Athl Train.* 2017;52(9):847-860.
24. Henriksen M, Creaby MW, Lund H, Juhl C, Christensen R. Is there a causal link between knee loading and knee osteoarthritis progression? A systematic review and meta-analysis of cohort studies and randomised trials. *BMJ Open.* 2014;4(7):e005368.
25. Henriksen M, Hunter DJ, Dam EB, et al. Is increased joint loading detrimental to obese patients with knee osteoarthritis? A secondary data analysis from a randomized trial. *Osteoarthritis Cartilage.* 2013;21(12):1865-1875.
26. Oiestad BE, Holm I, Risberg MA. Return to pivoting sport after ACL reconstruction: association with osteoarthritis and knee function at the 15-year follow-up. *Br J Sports Med.* 2018;52(18):1199-1204.
27. Alkjaer T, Simonsen EB, Jorgensen U, Dyhre-Poulsen P. Evaluation of the walking pattern in two types of patients with anterior cruciate ligament deficiency: copers and non-copers. *Eur J Appl Physiol.* 2003;89(3-4):301-308.
28. Alkjaer T, Simonsen EB, Peter Magnusson SP, Aagaard H, Dyhre-Poulsen P. Differences in the movement pattern of a forward lunge in two types of anterior cruciate ligament deficient patients: copers and non-copers. *Clin Biomech (Bristol, Avon).* 2002;17(8):586-593.
29. DeVita P, Aaboe J, Bartholdy C, Leonardis JM, Bliddal H, Henriksen M. Quadriceps-strengthening exercise and quadriceps and knee biomechanics during walking in knee osteoarthritis: A two-centre randomized controlled trial. *Clin Biomech (Bristol, Avon).* 2018;59:199-206.
30. Radin EL, Yang KH, Riegger C, Kish VL, O'Connor JJ. Relationship between lower limb dynamics and knee joint pain. *J Orthop Res.* 1991;9(3):398-405.
31. Culvenor AG, Ruhdorfer A, Juhl C, Eckstein F, Oiestad BE. Knee Extensor Strength and Risk of Structural, Symptomatic, and Functional Decline in Knee Osteoarthritis: A Systematic Review and Meta-Analysis. *Arthritis Care Res (Hoboken).* 2017;69(5):649-658.
32. de Jong SN, van Caspel DR, van Haeff MJ, Saris DB. Functional assessment and muscle strength before and after reconstruction of chronic anterior cruciate ligament lesions. *Arthroscopy.* 2007;23(1):21-28, 28 e21-23.
33. Birchmeier T, Lisee C, Kane K, Brazier B, Triplett A, Kuenze C. Quadriceps Muscle Size Following ACL Injury and Reconstruction: A Systematic Review. *J Orthop Res.* 2020;38(3):598-608.
34. Lisee C, Lepley AS, Birchmeier T, O'Hagan K, Kuenze C. Quadriceps Strength and Volitional Activation After Anterior Cruciate Ligament Reconstruction: A Systematic Review and Meta-analysis. *Sports Health.* 2019;11(2):163-179.
35. Astephen Wilson JL, Stanish WD, Hubley-Kozey CL. Asymptomatic and symptomatic individuals with the same radiographic evidence of knee osteoarthritis walk with different knee moments and muscle activity. *J Orthop Res.* 2017;35(8):1661-1670.

36. Henriksen M, Rosager S, Aaboe J, Graven-Nielsen T, Bliddal H. Experimental knee pain reduces muscle strength. *J Pain.* 2011;12(4):460-467.
37. Kellgren JH, Lawrence JS. Radiological assessment of osteo-arthrosis. *Ann Rheum Dis.* 1957;16(4):494-502.
38. Irrgang JJ, Anderson AF, Boland AL, et al. Development and validation of the international knee documentation committee subjective knee form. *Am J Sports Med.* 2001;29(5):600-613.
39. Roos EM, Lohmander LS. The Knee injury and Osteoarthritis Outcome Score (KOOS): from joint injury to osteoarthritis. *Health Qual Life Outcomes.* 2003;1:64.
40. Hawker GA, Davis AM, French MR, et al. Development and preliminary psychometric testing of a new OA pain measure--an OARSI/OMERACT initiative. *Osteoarthritis Cartilage.* 2008;16(4):409-414.
41. Tegner Y, Lysholm J. Rating systems in the evaluation of knee ligament injuries. *Clin Orthop Relat Res.* 1985(198):43-49.
42. Jaric S. Muscle strength testing: use of normalisation for body size. *Sports Med.* 2002;32(10):615-631.
43. Jorgensen TS, Hangaard S, Bliddal H, Henriksen M. Test-retest reliability of cuff pressure pain algometry in patients with knee osteoarthritis. *Clin Exp Rheumatol.* 2016;34(1):158.
44. Jespersen A, Dreyer L, Kendall S, et al. Computerized cuff pressure algometry: A new method to assess deep-tissue hypersensitivity in fibromyalgia. *Pain.* 2007;131(1-2):57-62.

Statistical analysis plan

STATISTICAL ANALYSIS PLAN: MIRAKOS

DETAILED STATISTICAL ANALYSIS PLAN (SAP)

1.0 ADMINISTRATIVE INFORMATION

1.1 TITLE, REGISTRATION, VERSIONS AND REVISIONS

Full study title	Musculoskeletal function in anterior cruciate ligament reconstructed individuals with and without knee pain.
Acronym	MIRAKOS
Local project number	APPI2-PT-2020-02
Ethics committee number	H-20060332
Study protocol version	1.2 (6 December 2021)
SAP version	1.0 (23 February 2022)

SAP revision history

Version #	Issue date	Amendment

SAP revision justification	-
SAP revision timing	-

1.2 ROLES AND RESPONSIBILITY

Author	Tine Alkjær[1,2]
Statistician	Marius Henriksen[2]
Principle investigator	Tine Alkjær[1,2]
Contributors and roles	Elisabeth Bandak[1,2], contributing to design and measurements, SAP revision.<br>Marius Henriksen[2], statistics, contributing to design, SAP revision.<br>Lauri Stenroth[3], contributing to design, measurements and outcome calculation, SAP revision.
Affiliations	[1]Department of Biomedical Sciences, University of Copenhagen, Copenhagen, Denmark.<br>[2]The Parker Institute, Bispebjerg-Frederiksberg Hospital, Copenhagen, Denmark.<br>[3]Department of Applied Physics, University of Eastern Finland, Kuopio, Finland.

1.3 SIGNATURES

We the undersigned, certify that we read this SAP and approve it as adequate in scope of the main analyses of the MIRAKOS.

1.3.1 Author

Name: Tine Alkjær

……………………………………………………

Date:

1.3.2 Statistician

Name: Marius Henriksen

……………………………………………………

Date: 23/02/2022

1.3.3 Principle investigator

Name: Tine Alkjær

……………………………………………………

Date:

2.0 INTRODUCTION

2.1 BACKGROUND AND RATIONALE

Knee osteoarthritis (OA) is the most common joint disease and a significant contributor to global disability [1]. The known knee OA risk factors include obesity, surgery, occupational load and injury [2,3]. Anterior cruciate ligament (ACL) rupture is a common knee injury [4,5] and the incidence is increasing, particularly among young people [6]. ACL injury affects the knee joint function and increases the risk of knee OA development [7-10] even at a young age, which prolongs the period of impaired function and pain [11]. Most research has focused on radiographic knee OA while fewer studies have investigated the prevalence of symptomatic knee OA after ACL injury [12]. It is important to discriminate between radiographic and symptomatic knee OA, as knee pain is a decisive criterion to diagnose knee OA [13], whereas radiographic changes serve more as a confirmatory measure. Indeed, the Framingham study showed that the prevalence of radiographic changes (indicative of OA) in the population older than 63 years was 33% whereas the prevalence of symptoms was only 9% [14]. A recent MRI study of 230 asymptomatic knees reported that 97% of these showed abnormalities in at least one knee structure [15]. This emphasizes that image-based signs of knee OA are not always accompanied by pain and OA symptoms.

Conventionally, mechanical joint loading is proposed as a key mechanism contributing to the development and progression of OA [16,17]. Thus, the knee joint loading during dynamic tasks in the ACL injured population has been studied extensively due to the supposed link between the knee joint compressive forces and the onset of post-traumatic knee OA [18-23]. However, the evidence for a causal link between knee joint loading and knee OA development and progression is weak [24,25]. Furthermore, a 15-year follow-up study, showed that ACL reconstructed persons returning to pivoting sport (presumably associated with high and multidirectional loads) had reduced odds of developing knee OA and had a better self-reported function in activities of daily living [26]. On the other hand, data suggest that ACL reconstructed individuals develop different adaptive neuromuscular functions [27,28], and it is possible that other mechanical factors than loading magnitude are implicated in the development of knee OA. Such other biomechanical factors may include force dissipation capacity of the musculoskeletal system [29], micro-incoordination [30], muscle strength and other aspects of muscle function. Low quadriceps muscle strength is associated with an increased risk of worsening symptoms and functional deterioration in people with and at risk of radiographic knee OA [31]. The quadriceps muscle strength and function are impaired after ACL injury and strength deficits persist even after ligament reconstruction [32-34]. Altogether, there are indices and a common agreement that poor musculoskeletal function is associated with increased risk of development of both symptomatic and radiographic knee OA, and that an ACL injury and reconstruction may lead to unfavourable changes in the musculoskeletal function accelerating the development of symptoms and/or degenerative OA changes. One study has compared individuals with definitive radiographic OA with and without symptoms and found that the symptomatic group had lower muscle strength and walking biomechanics indicative of a "stiffer" gait, possibly reflecting protective neuromuscular adaptations in the walking pattern [35]. As ACL injuries increase the risk of OA (symptomatic and radiographic) later in life, the musculoskeletal function may be changed alongside the early onset of symptoms but before definitive radiographic OA is present. Thus, the present study will compare the musculoskeletal function between ACL reconstructed individuals with and without knee pain. By this, we can deepen our understanding of the role of musculoskeletal function in relation to the development and progression of knee OA.

2.2 OBJECTIVES

2.2.1 Objectives and research questions

The objective of the present study is to compare the musculoskeletal function between ACL reconstructed individuals with and without knee pain to answer the research question: Are there differences in the musculoskeletal function in ACL reconstructed individuals with knee pain when compared to those without knee pain?

The musculoskeletal function will be assessed by

- Muscle strength of the knee extensor muscle (quadriceps)
- Biomechanics of the knee and quadriceps muscle during walking and a forward lunge movement

2.2.2 Hypotheses

1) ACL reconstructed individuals without knee pain have stronger quadriceps muscles compared to those with knee pain.
2) ACL reconstructed individuals without knee pain develop higher quadriceps muscle forces and knee joint loading during walking and forward lunging compared to those with knee pain.

2.2.3 Scope

This SAP is structured as recommended for observational studies[36]. It will be the guiding document for the main analyses testing the two hypotheses and will exclusively include outcomes obtained from the ACL reconstructed leg (see section 6.1.1).

The data obtained from the participants' contralateral (i.e. non-operated) leg will be reported in subsequent sub-studies with separate SAP documents.

3.0 STUDY METHODS

3.1 GENERAL STUDY DESIGN AND PLAN

This is a cross-sectional observational study. Two groups of ACL reconstructed individuals identified as symptomatic (with knee pain) and asymptomatic (without knee pain) are invited to participate in the study that takes place at The Parker Institute/Bispebjerg-Frederiksberg Hospital, Copenhagen, Denmark. The study protocol (APPI2-PT-2020-02) was written and approved by the local ethics committee before study initiation. The final protocol (version 1.2) is published on the Parker Institute's website. This SAP was written after the initiation of the data collection (June 2021) and finalized before the inclusion of the last participant. The author of the SAP was not blinded to the database during writing the SAP. However, data were not summarized or analyzed before completing the SAP.

3.2 SAMPLE SIZE, POWER AND DETECTABLE DIFFERENCE

We will compare the quadriceps muscle strength between two groups of ACL reconstructed individuals: 1) symptomatic and 2) asymptomatic.

The variance in this population is unknown, while our sample size estimation will be pragmatic. To detect a group difference of 0.3 Nm/kg in the primary outcome with a common standard deviation of 0.5 Nm/kg, a sample size of 50 per group will have a power of 84%, ($\alpha = 0.05$). Thus, a total sample size of n=100 (50/50 per group) was originally intended to be applied. However, in November 2021 71 (17/54 symptomatic/asymptomatic) participants were recruited and included in the study, indicating a low prevalence of symptomatic participants. We estimated that it would be difficult to recruit

50 symptomatic participants within the time allotted for recruitment (scheduled to last until August 2022). In contrast, the recruitment of participants to the asymptomatic group has proven to be efficient. Therefore, we decided to re-calculate the sample size and change the group allocation ratio from 1:1 to 1:3. To detect that same group difference with the same common standard deviation of the primary outcome as stated above, a new total sample size was estimated to n=120 (n=30 symptomatic and n=90 asymptomatic participants) with a statistical power of 80.6%. This amendment was registered in the study protocol 4th November 2021. Currently, our goal is to continue the data collection until the inclusion of 120 eligible participants (symptomatic/asymptomatic; n=30/90) is reached. However, the inclusion of participants will end in August 2022, leaving some uncertainty about the final sample size.

3.3 TIMING OF FINAL ANALYSIS

The final analysis will be done after 31/8 2022 but no later than 31/12 2022 and presupposes that the inclusion of participants has ended, all data have been collected and the database has been closed. The SAP will be published on the Parker Institute's website along with the study protocol before any data analyses are conducted.

3.4 TIMING OF OUTCOME ASSESSMENTS

The processing of motion capture data and musculoskeletal modelling to provide biomechanical variables is time-consuming and will be done after the measurement visit of each participant. Thus, the outcomes to assess the knee and quadriceps muscle function during walking and forward lunge movement are generated continuously and registered in the database during the data collection period. This will continue for a short period after the inclusion of the last participant. All other outcomes and data are registered in the database at or immediately after the measurement visit. The participants fill out electronic-format questionnaires for assessment of self-reported knee function at the measurement visit and these data are registered directly in the database. While the research team will not be blinded to the data, there will not be any data extraction before the data collection has ended/the database closed and the SAP has been finalized.

4.0 STATISTICAL PRINCIPLES

4.1 MULTIPLICITY

We will not adjust for multiplicity. We are fully aware that the risk of type I error is present as we will make many comparisons and our outcomes are very likely correlated. Thus, we will explicitly state that the analyses are exploratory and hypothesis-generating and that the results may need replication in studies with a more causal design.

4.2 STATISTICAL SIGNIFICANCE AND CONFIDENCE INTERVALS

A P-value < 0.05 are considered statistically significant for our primary outcome. Results will be reported as mean values with standard deviation (SD), or group mean differences with a 95% confidence interval (CI).

4.3 ADHERENCE AND PROTOCOL DEVIATIONS

4.3.1 Definitions of protocol deviations

Protocol deviations are defined as study activities that diverge from the local institutional review board reviewed protocol but without significant consequences[37].

4.3.2 Protocol deviations to be summarised

The following deviations from the protocol have been identified:

- The pressure pain sensitivity was assessed by *two* pressure pain thresholds: 1) the pressure pain detection threshold (PDT) and 2) the pressure pain tolerance threshold (PTT). The PTT defines the pressure, at which the pain becomes intolerable (section 6.2.7).
- The current knee pain was assessed by a verbal rating scale (VRS) 0-10 *both* during walking/forward lunging *and* during the muscle strength testing, meaning that after each walking/lunging/contraction trial the participant verbally rated the current knee pain on a 0-10 VRS (section 6.2.9).
- The knee joint laxity testing was part of the clinical examination *but* an instrumented knee joint laxity test was also done during the experiments to quantify the laxity and report it as a participant characteristic (section 6.2.10).
- The second hypothesis was originally formulated: "ACL reconstructed individuals without knee pain develop higher quadriceps muscle forces and knee joint compressive forces during walking and forward lunging compared to those with knee pain". This has been refined: "ACL reconstructed individuals without knee pain develop higher quadriceps muscle forces and knee joint loading during walking and forward lunging compared to those with knee pain". The reason for this change is that we assess *both* the knee joint compressive force *and* the knee extensor moment and these two parameters are covered under "knee joint loading".

The above was either not explicitly stated in the study protocol or reflects refinements to its content.

This SAP focus on measurements from the ACL reconstructed leg while observations from the contralateral leg will be reported in subsequent sub-studies (section 2.2.3).

5.0 STUDY POPULATION

5.1 SCREENING DATA

Screening data were collected with the purpose to describe the eligibility of all potential participants responding positively to the study invitation letter (see section 5.3). Thus, reasons for non-eligibility will be documented, and includes (but are not limited to):

- Major surgery to the other knee, e.g., ACL reconstruction.
- Other musculoskeletal pain in the lower extremities.
- BMI > 30.
- Neuromuscular diseases.

5.2 ELIGIBILITY

The aim was to compare participants with and without knee pain, the eligibility criteria were as follows:

Participants with knee pain ("Symptomatic group"):

Inclusion criteria:

- Age between 18 and 40 years at the time of ACL reconstruction.
- Primary ACL reconstruction using the semitendinosus-gracilis tendon graft.
- Post-surgery time of at least 3 years.

- Current body mass index (BMI) of ≤30.
- Pain score of at least 3 (VRS 0-10) in the reconstructed knee during activities of daily living (ADL) within the last week.

Participants without knee pain ("Asymptomatic group"):

Inclusion criteria:

- Age between 18 and 40 years at the time of ACL reconstruction.
- Primary ACL reconstruction using the semitendinosus-gracilis tendon graft.
- Post-surgery time of at least 3 years.
- Current body mass index (BMI) of ≤30.
- Pain score of 0 (VRS 0-10) in the reconstructed knee during activities of daily living (ADL) within the last week.

For both groups, the exclusion criteria are the same:

Exclusion criteria:

- Known neuromuscular diseases.
- Evidence of cartilage lesions ICRS grade 4 (full thickness) from MRI at time of ACL reconstruction or documented peri-surgically.
- ACL reconstruction or other major surgery to the other knee.
- Congenital deformities in the lower extremities preventing full participation in the tests.
- Current musculoskeletal pain in other regions of the lower extremity other than the injured knee.
- Any other condition that in the opinion of the investigator makes a potential participant unfit for participation or conditions that puts a potential participant at risk by participation.

5.3 RECRUITMENT

ACL reconstructed persons were identified in the Danish Ligament Reconstruction (DLR) Register and invited to participate in the study by sending them an invitation letter via digital mail (e-Boks) stating the main criteria for participation. A flow diagram will be used to visualize the flow of participants. Here we will report the population identified in the DLR register and from where eligible participants were selected, reasons for exclusions and how many were included and allocated to the symptomatic and asymptomatic group and any withdrawals. See figure 1 for an example.

5.4 WITHDRAWAL/FOLLOW-UP

This study is a cross-sectional observational study with no interventions applied. Thus, we expect the withdrawal rate to be negligible. Withdrawal can occur when the eligible participants refuse to participate or if other issues emerge preventing the participant from participating in the experiments after written informed consent has been obtained.

5.5 BASELINE PARTICIPANT CHARACTERISTICS

5.5.1 Collected baseline participant characteristics

Most of the data are collected at the measurement visit (one day) while only a few clinical data are extracted from electronic registry databases (e.g. DLR Register or patient record via the electronic medical journal "Sundhedsplatformen" (SP)) and few participants characteristics are registered during screening. Table 1 displays an overview of all the collected variables.

5.6 ASSUMED CONFOUNDING COVARIATES

Although, we cannot exclude that our measured variables may be influenced by measured and unmeasured variables (e.g. genetic, environmental, psychological) that potentially confound the interpretation of the results leading to wrong conclusions, we have not been able to identify any covariates that clearly would influence *both* the exposure (presence of knee pain) *and* outcome (musculoskeletal function). Thus, no adjustments for confounding covariates will be applied in our statistical analyses.

6.0 ANALYSIS

6.1 OUTCOME DEFINITIONS

The analysis of our primary and secondary outcomes shall answer the research question and test the two hypotheses (see section 2.2).

6.1.1 Study knee

The study knee is defined as the knee at which the ACL was reconstructed. The outcomes are obtained from the participants' study knee and used as input parameters to the statistical analyses (section 6.3).

6.1.2 Primary outcome

The primary outcome is the maximal isometric quadriceps muscle strength defined as the highest torque value measured among three separate maximal voluntary isometric contraction (MVIC) repetitions. The unit for the primary outcome is Nm/kg (see section 6.2.1).

6.1.3 Key secondary outcomes

The following outcomes are assessed as key secondary outcomes:

- The peak knee extensor moment during walking defined as the mean of the individual peak knee extensor moment values across six walking trials. The unit for this outcome is Nm/kg (section 6.2.2).
- The peak knee extensor moment during the forward lunge defined as the mean of the individual peak knee extensor moment values across three forward lunge trials. The unit for this outcome is Nm/kg (section 6.2.2).
- The peak quadriceps muscle force during walking defined as the mean of the individual peak quadriceps muscle force values across six walking trials. The unit for this outcome is N/kg (section 6.2.2).
- The peak quadriceps muscle force during the forward lunge defined as the mean of the individual peak quadriceps muscle force values across three forward lunge trials. The unit for this outcome is N/kg (section 6.2.2).
- The peak knee joint contact force during walking defined as the mean of the individual peak knee joint contact force values across six walking trials. The unit for this outcome is N/kg (section 6.2.2).
- The peak knee joint contact force during the forward lunge defined as the mean of the individual peak knee joint contact force values across three forward lunge trials. The unit for this outcome is N/kg (section 6.2.2).

6.1.4 Other secondary outcomes

The following outcomes are assessed as other secondary outcomes:

- The maximal isometric hamstring muscle strength defined as the highest torque value measured among three separate MVIC repetitions. The unit for the primary outcome is Nm/kg (section 6.2.1).
- The five Knee Injury and Osteoarthritis Outcome Score (KOOS) subscales (pain; symptoms; function in activities of daily living; function in sports and recreational activity; knee-related quality of life (QoL) (section 6.2.3).
- The International Knee Documentation Committee (IKDC) score (section 6.2.4).
- The intermittent and constant osteoarthritis pain (ICOAP) total score and two subscales: the constant pain subscale and intermittent pain subscale (section 6.2.5).
- The change in Tegner scores from the pre-injury activity level to the current activity level (section 6.2.6).
- The pressure pain detection threshold (PDT) defined as mean of the three measurements. The unit for this outcome is kPa, (section 6.2.7).
- The pressure pain tolerance threshold (PTT) defined as mean of the three measurements. The unit for this outcome is kPa, (section 6.2.7).
- The Kellgren and Lawrence grading scale (the radiographic knee OA level) (section 6.2.8).
- The peak knee flexion angle in the first half of the stance phase during walking defined as the mean of the individual peak knee flexion angle values across six walking trials. The unit for this outcome is ° (section 6.2.2).
- The peak knee flexion angle during the forward lunge defined as the mean of the individual peak knee flexion angle values across three forward lunge trials. The unit for this outcome is ° (section 6.2.2).
- The walking speed defined as the mean of the individual speeds across six walking trials. The unit for this outcome is m/s (section 6.2.2).
- The forward lunge foot-ground contact time defined as the mean of the individual time duration of foot-ground contact across three forward lunge trials. The unit of this outcome is s (section 6.2.2).
- The current knee pain during walking defined as the mean of the individual VRS scores reported during six walking trials (section 6.2.9).
- The current knee pain during the forward lunge defined as the mean of the individual VRS scores reported during three forward lunge trials (section 6.2.9).
- The current knee pain during the quadriceps muscle strength test defined as the mean of the individual VRS scores reported after three MVIC trials (section 6.2.9).
- The current knee pain during the hamstring muscle strength test defined as the mean of the individual VRS scores reported after three MVIC trials (section 6.2.9).

6.2 MEASUREMENTS AND CALCULATION OF OUTCOMES

6.2.1 Muscle strength

MVICs of the quadriceps and hamstring muscle strength will be assessed using an isokinetic dynamometer (Biodex System4 Pro, Biodex Medical System, NY, USA) at 60° knee flexion. The participants are seated in a rigid chair firmly strapped to the seat across the chest, at the hip and distal thigh. The rotation axis of the dynamometer is visually aligned to the lateral femoral epicondyle and the lower leg attached to the lever arm of the dynamometer. The lever arm is placed just above the lateral malleolus and firmly fixed with a cuff. The participants are asked to perform the MVICs with maximal effort and verbal encouragement will be provided during testing that comprises three repetitions

of which the highest peak torque value defines the maximal quadriceps/hamstring muscle strength and will be reported as body mass normalized values (Nm/kg)[38].

6.2.2 Biomechanics modelling and simulation

Experimental data

Anthropometric parameters required for scaling the biomechanical model are obtained from the participants. Participants are fitted with 39 reflective markers. A static standing calibration and functional calibration movements for the hip joint centre (star-arc) and knee joint axis (half squat) are first performed. Then the participants perform walking with self-select walking speed and forward lunges in the motion capture laboratory. The instruction in the forward lunge is to take a long step forward, go down to 90 degrees of knee flexion and return to the standing posture as fast as possible. During the movements marker trajectories (100 Hz, Vicon Motion Systems Ltd, UK) and ground reaction forces (1000 Hz, OR-6, Advanced Mechanical Technology Inc., USA) will be recorded. Gaps in the marker trajectories are filled and marker trajectories and ground reaction forces data are low-pass filtered (recursive 4th order low-pass Butterworth filter with 6Hz cut-off frequency[39]). Six walking trials all within ±0.1 km/h and three forward lunges are selected for further analysis using musculoskeletal modelling and simulation.

Musculoskeletal modelling and simulation

Musculoskeletal modelling simulation is performed in OpenSim software[40]. A musculoskeletal model designed for analysis of movement with large hip and knee joint excursions is used[41]. The model is further modified to improve knee extensor muscle moment arm at large knee flexion angles and to account for autograft donor muscle impairment by adjusting semitendinosus and gracilis muscle optimal fibre length and maximal isometric muscle force as per Saxby et al. (2016)[42]. Then, the model is scaled to match the mass and dimensions of the participant based on the measured body mass and marker locations from a standing calibration trial. The estimated hip joint centre[43] and knee joint axis[44] are used to assist scaling of the pelvis, femur, and tibia segments and subsequently to locate joint centres. The maximum isometric force of the knee extensor and flexor muscles are scaled to match experimentally measured knee extension and flexion strength, respectively. In all other muscles, it is assumed that the muscle strength scales relative to the body mass of the participants. Joint kinematics are calculated using inverse kinematics algorithm in OpenSim followed by inverse dynamics to calculate intersegmental resultant forces and moments. Static optimization is used to estimate muscle forces while accounting for muscle force-length properties with a cost function minimizing the sum of squared muscle activations[45]. Finally, a joint reaction analysis tool is used to estimate knee joint contact forces. The simulations are performed for the ground contact phase of walking and forward lunge.

The biomechanical outcomes are means of six successful walking trials/three successful forward lunge trials. The outcomes of the walking trials (peak knee extensor moment, peak quadriceps muscle force, peak knee joint contact force, peak knee flexion angle) are extracted from the 1st half of the stance phase. We focus on the 1st half of the stance phase since in this phase it is the knee extensor muscles that are controlling the knee flexion and are the main contributors of the compressive forces at the tibiofemoral joint. Later in the stance, the main contributor to the compressive force is gastrocnemius muscles [46]. Regarding the forward lunge trials these outcomes are extracted across the whole foot-ground contact phase. The peak knee extensor moment, peak quadriceps muscle force, peak knee joint contact force outcomes will be normalized to body mass; moments are expressed as Nm/kg and forces as N/kg.

The walking speed is assessed by photocells during each individual walking trial and calculated as the mean walking speed (m/s) of the six walking trials selected for further biomechanical analysis.

The forward lunge foot-ground contact time is assessed as the time in s where the foot is in contact with the force plate during the lunge movement. The vertical ground reaction force signal is used to detect this period. The foot is considered to be in contact with the ground when the ground reaction force signal is above 10 N. The forward lunge foot-ground contact time outcome is calculated as the mean of the three forward lunge trials selected for the biomechanical analyses.

6.2.3 KOOS

The Knee injury and Osteoarthritis Outcome Score (KOOS), a disease-specific instrument, is an extension of the Western Ontario and McMaster Universities Osteoarthritis Index (WOMAC) [47]. The KOOS consists of 42 items covering five domains, namely, Pain (9 items), Symptoms (7 items), Activities of Daily Living (ADL) (17 items), Sports and Recreation (5 items), and knee-related QoL (4 items). The KOOS adopts a five-point Likert scale scoring system (ranging from 0 (least severe) to 4 (most severe). A normalized score is calculated for each domain with 100 indicating no symptoms and functional impairment and 0 indicating extreme symptoms and functional impairment. In accordance with the user guide (http://www.koos.nu), if the number of missing items is less than or equal to 2 in a subscale, they will be substituted by the average item value for that subscale. If more than two items of the subscale are omitted, the response will be considered invalid, and no subscale score calculated.

6.2.4 The International Knee Documentation Committee (IKDC questionnaire)

The IKDC questionnaire is an instrument to assess patients with a variety of knee disorders including ligamentous and meniscal injuries as well as patellofemoral pain and osteoarthritis[48]. The questionnaire consists of three subscales: symptoms (7 items), sports activity (2 items), and knee function (2 items) and provides an overall function score. The scores are obtained by summing the individual items and then converting the crude total to a scaled number that ranges from 0 to 100. This final number represents a measure of function with higher scores representing higher levels of function. Thus, a score of 100 reflects no functional limitations. The IKDC score may be calculated if there are missing data, providing that responses have been given for at least 90% of the items. To calculate the IKDC score in case data are missing, the average score of the items that have been answered will be used to substitute for the missing item score(s). In case responses are missing for more than 90% of the items the IKDC response will be considered invalid.

6.2.5 Intermittent and Constant Osteoarthritis Pain questionnaire (ICOAP)

The ICOAP is a diagnosis-specific 11-item questionnaire designed to assess the pain experienced within the last week among people suffering from knee and hip OA[49]. The questionnaire is divided into two domains, a 5-item scale for constant pain and a 6-item scale for intermittent pain (so-called ''pain that comes and goes''). Each domain captures pain intensity as well as related distress and the impact of OA pain on quality of life. All items are scored on anchored rating scales with five levels of response (0–4). The ICOAP outcomes comprise the two subscales 1) the constant pain subscale (0–20) and 2) the intermittent pain subscale (0–24), and the total pain score (0–44). Normalized scores for the two subscales and the total pain score, from 0 (best) to 100 (worst), are calculated. If there are three or more items missing, the response is considered invalid. If there are less than 3 items missing,

the missing item can be replaced with the mean of the responses to other items within the same sub-scale[1].

6.2.6 Tegner score

The Tegner activity scale is an instrument to measure activity following knee injuries[50]. It grades activity based on work and sports activities on a scale of 0 to 10 one-item scores. Zero represents disability due to knee problems and 10 represents competitive sports (e.g., soccer - national and international elite level). The subjects report the level of participation that best describes their current level of activity and that before the injury. The change in Tegner score is calculated as the difference between the current activity level Tegner score and pre-injury activity level Tegner score. Negative values will indicate a decline in the activity level.

6.2.7 Pressure pain sensitivity

The pain sensitivity will be assessed by computerised cuff pressure algometry (CPA)[51]. A double-chambered Tourniquet cuff is wrapped around the calf by the gastrocnemius muscles of the lower extremity of the ACL reconstructed leg. A computer-controlled compressor inflates the cuff with air at 1 kPa/s[52]. The participant is asked to indicate the first sensation of pain on the handheld device with a slider by moving the slider upwards (lowest level indicate no pain, highest level indicate worst imaginable pain). The inflation continues until the participant presses the stop button on the handheld device. The pressure pain sensitivity is assessed by two pressure pain thresholds: the pressure pain detection threshold (PDT) and the pressure pain tolerance threshold (PTT). The PDT defines the pressure where the pain is detected (i.e., the first time the participant moves the slider away from zero) and PTT defines the pressure, at which the pain becomes intolerable (i.e., where the participant presses the stop button). The recorded pressures are measured in kPa. The test is repeated four times separated by resting periods of 3 minutes. The first measurement is used for familiarization and the following three are used for the analysis. The PDT and PTT outcomes are calculated as means of the three measurements.

6.2.8 The radiographic knee OA level

The evaluation of radiographic signs of knee OA is done according to Kellgren-Lawrence grading[53] at Frederiksberg Hospital by the same highly experienced rheumatologist. Scores on the Kellgren–Lawrence scale range from 0 to 4, with a score of 2, 3, or 4 indicating definite osteoarthritis and higher scores indicating more severe disease.

6.2.9 Knee pain during movement/muscle strength tests

The current knee pain during the muscle strength, walking and forward lunge tests will be assessed by a VRS 0-10 immediately after each trial, where 0 indicates 'no pain at all' and 10 indicates 'worst imaginable pain'. The knee pain will be calculated as the mean VRS reported during the trials selected for further analysis (for walking this is six and for forward lunge/muscle strength, it is three trials).

6.2.10 Knee joint laxity

Instrumented knee joint laxity testing will be measured using a digital arthrometer (Lachmeter, Lachmeter Company Equipamentos Ortopedicos LTDA, Ribeirao Preto, Brazil). This quantifies the anterior translation of the tibia relative to the femur. The participant is lying supine on an examination table with a wedge cushion behind the thigh ensuring the same degree of knee flexion for all measurements. The participant is asked to relax the thigh muscles and especially the hamstrings during the

[1] https://oarsi.org/sites/default/files/docs/2013/icoap_users_guide_07072010.pdf

test. The knee joint laxity will be assessed three times for each knee. The mean of the three assessments for each leg are calculated and the knee joint laxity side-to-side difference will be calculated by subtracting the mean knee joint laxity of the contralateral knee from the mean knee joint laxity of the ACL-reconstructed knee. The unit of this variable is mm. The knee joint laxity is included in the participant characteristics (Table 2).

6.3 STATISTICAL ANALYSIS METHODS

6.3.1 Primary analysis

The primary analysis applied for estimation of between-group differences of all selected outcomes (section 6.1) will be an analysis of covariance (ANCOVA) for continuous data. The results will be reported as mean ± SD, mean differences with 95% confidence interval (CI) and the level of significance is set to 0.05.

Categorical data and counts (percentages) will be analysed using Chi-square statistics comparing distributions between groups.

Binary data will be presented as risk differences with 95% CI.

The results of the analyses will be presented in tables that resemble the Tables 2-4 shown in section 9.0.

6.4 MISSING DATA

6.4.1 Reasons for missing data

Missing data may potentially occur due to technical issues, electrical power supply breakdown, or other unforeseen issues related to the test equipment. If this should happen the missing data will be considered as missing completely at random.

6.4.2 Imputation method

In case, a given variable has more than 10% missing data, multiple imputation will be applied. All the existing data of that variable will be used to predict the missing values.

6.5 STATISTICAL SOFTWARE

The analyses are done using the statistical software SAS version 9.4 (SAS Institute Inc., Cary, NC, USA).

7.0 DISCUSSION

The overall aim of the MIRAKOS study is to compare the musculoskeletal function between ACL reconstructed individuals with and without knee pain to answer the research question: Are there differences in the musculoskeletal function in ACL reconstructed individuals with knee pain when compared to those without knee pain? Specifically, we will test two hypotheses:

1) ACL reconstructed individuals without knee pain have stronger quadriceps muscles compared to those with knee pain.
2) ACL reconstructed individuals without knee pain develop higher quadriceps muscle forces and knee joint loading during walking and forward lunging compared to those with knee pain.

The rationale for the hypotheses is based on research documenting that quadriceps muscle weakness is associated with an increased risk of worsening symptoms and functional deterioration in people

with and at risk for radiographic knee OA[31,35], and that knee joint pain has a negative impact on quadriceps muscle activation and force production[54].

Although, the aim is to test these hypotheses we are fully aware of the fact that the cross-sectional study design will render our analyses exploratory. We will be able to describe and quantify possible differences in the musculoskeletal function between the two study groups; and then likely generate new hypotheses that may be investigated in future studies designed to determine causations.

8.0 CONCLUSION

This SAP presents the basis for the analyses and outcome selection in the MIRAKOS cross-sectional study and discusses the methodological and statistical concerns associated with it. We aim to report the results of the MIRAKOS study as transparently and clearly as possible in order to mitigate outcome reporting bias and data-driven results.

9.0 TABLES AND FIGURES

Figure 1. Flow diagram (example)

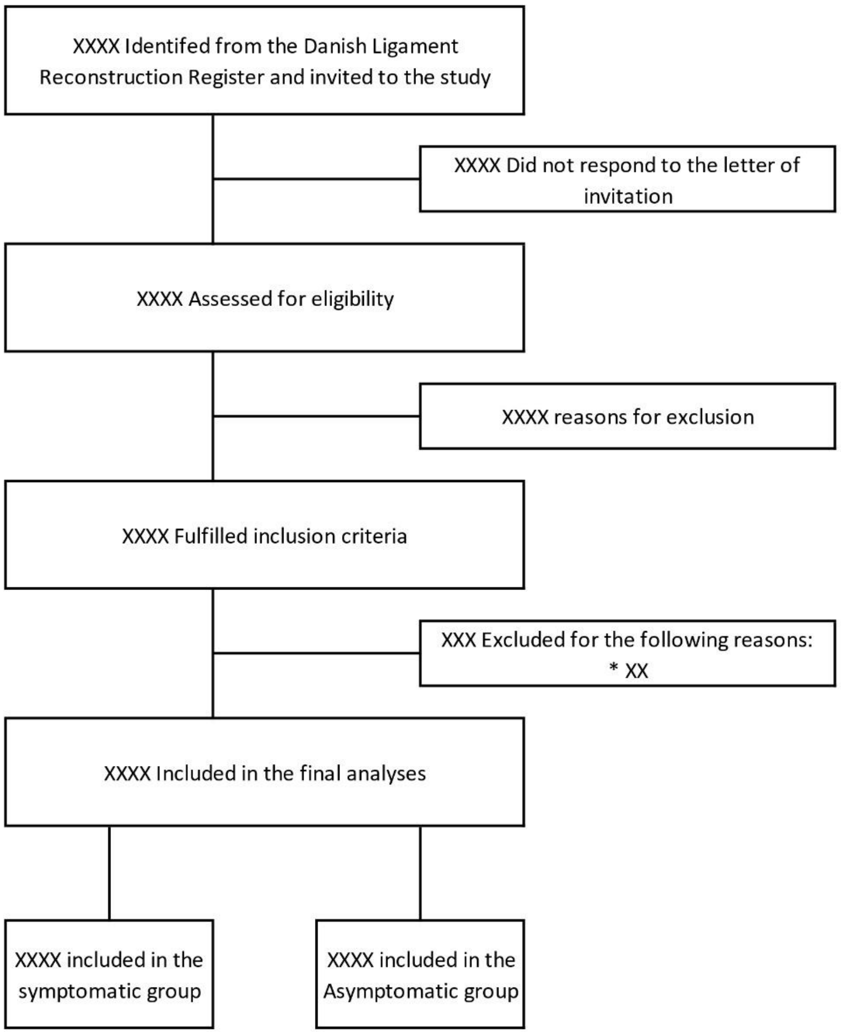

Table 1. Overview of all measured variables.

Variable	At study visit	Not at study visit (tool)
**Demographic/Clinical**		
Age	X	
Sex	X	
Height	X	
Weight	X	
Body mass index	X	
Injury situation	X	
Injured knee (side)	X	
Reconstruction graft type		X (DLR Register)
Time since surgery (month)		X (DLR Register)
Knee joint laxity	X	
Radiographic knee OA level (K-L score)	X	
**Questionnaires**		
Pre-injury activity level (Tegner score)	X	
Current activity level (Tegner score)	X	
Knee function (IKDC)	X	
Knee function (KOOS)	X	
Knee pain experience (ICOAP)	X	
**Muscle strength**		
Maximal isometric quadriceps strength	X	
Maximal isometric hamstring strength	X	
**Movement biomechanics**		
Walking speed	X	
Peak knee extensor moment (walking/forward lunge)		X (musculoskeletal modelling)
Peak knee flexion angle (walking/ forward lunge)		X (musculoskeletal modelling)
Forward lunge movement time		X (musculoskeletal modelling)
Peak quadriceps muscle force (walking/ forward lunge)		X (musculoskeletal modelling)
Peak knee joint contact force (walking/ forward lunge)		X (musculoskeletal modelling)
**Knee pain**		
VRS during muscle strength tests	X	
VRS during walking/forward lunge tests	X	
**Pressure pain sensitivity**		
Pressure pain detection threshold (PDT)	X	
Pressure pain tolerance threshold (PTT)	X	

Table 2. Participant characteristics

**Variable**	Asymptomatic (N=xx)	Symptomatic (N=xx)	Estimated difference	P-value
**Demographic/Clinical**				
Age, years				
Male sex, no. (%)				
Height, m				
Body mass, kg				
Body mass index, kg/m^2				
Injured knee (right), no. (%)				
Reconstruction graft type				
Time since surgery, month				
Knee joint laxity, mm				
**Injury situation*, no. (%):**				
Traffic accident				
Sport injury				
Other				
**Type of sport*, no. (%)**				
Team ball sports				
Racket sports				
Martial arts				
Other				
**Return to sport*, no. (%)**				
No				
Yes				
Partly#				
**Radiographic knee OA level (K-L score)$, no. (%)**				
0				
1				
2				
3				
4				
**Activity level**				
Pre-injury activity level (Tegner score)				
Current activity level (Tegner score)				

*Obtained during screening interview.
#Meaning “yes but not at the same pre-injury level”.
$Scores on the Kellgren–Lawrence scale range from 0 to 4, with a score of 2, 3, or 4 indicating definite osteoarthritis and higher scores indicating more severe disease.

Table 3. Group means (SD) and mean differences (95% CI) of muscle strength, walking and forward lunge knee biomechanics, knee pain during movement/muscle strength tests and pressure pain sensitivity variables including statistical probability.

	Asymptomatic (N=xx)	Symptomatic (N=xx)	Estimated difference	P-value
	Mean (SD)	Mean (SD)	Group Mean Difference (95% CI)	
Muscle strength				
Maximal isometric quadriceps muscle strength (Nm/kg)*				
Maximal isometric hamstring muscle strength (Nm/kg)ǂ				
Walking biomechanics				
Peak knee extensor moment during walking (Nm/kg)§				
Peak quadriceps muscle force during walking (N/kg)§				
Peak knee joint contact force during walking (N/kg)§				
Peak knee flexion, walking (°)ǂ				
Walking speed, (m/s)ǂ				
Forward lunge biomechanics				
Peak knee extensor moment during forward lunge (Nm/kg)§				
Peak quadriceps muscle force during forward lunge (N/kg)§				
Peak knee joint contact force during forward lunge (N/kg)§				
Peak knee flexion, forward lunging (°)ǂ				
Forward lunge foot-ground contact time (s)ǂ				
Pain during movement/muscle strength tests				
Current knee pain during walkingǂ				
Current knee pain during forward lungeǂ				
Current knee pain during quadriceps muscle strength testǂ				
Current knee pain during hamstring muscle strength testǂ				
Pressure pain sensitivity				
Pressure pain detection threshold (kPa)ǂ				
Pressure pain tolerance threshold (kPa)ǂ				
*Primary outcome measure; §Key secondary outcome measures; ǂOther secondary outcome measures				

Table 4. Group means (SD) and mean differences (95% CI) of questionnaires (patient reported outcomes) and activity level variables including statistical probability.

	Asymptomatic (N=xx)	Symptomatic (N=xx)	Estimated difference	P-value
	Mean (SD)	Mean (SD)	Group Mean Difference (95% CI)	
Questionnairesǂ				
KOOS Pain score				
KOOS Symptoms score				
KOOS Quality of life score				
KOOS Sports and recreation score				
KOOS Quality of life score				
International Knee Documentation Committee score				
ICOAP Total score				
ICOAP Constant Pain subscore				
ICOAP Intermittent Pain subscore				
Activity levelǂ				
Change in Tegner score activity level (current - preinjury)				
ǂOther secondary outcome measures				

10.0 REFERENCES

1. Cross M, Smith E, Hoy D, et al. The global burden of hip and knee osteoarthritis: estimates from the global burden of disease 2010 study. *Ann Rheum Dis.* 2014;73(7):1323-1330.
2. Felson DT. Clinical practice. Osteoarthritis of the knee. *N Engl J Med.* 2006;354(8):841-848.
3. Blagojevic M, Jinks C, Jeffery A, Jordan KP. Risk factors for onset of osteoarthritis of the knee in older adults: a systematic review and meta-analysis. *Osteoarthritis Cartilage.* 2010;18(1):24-33.
4. Kaeding CC, Leger-St-Jean B, Magnussen RA. Epidemiology and Diagnosis of Anterior Cruciate Ligament Injuries. *Clin Sports Med.* 2017;36(1):1-8.
5. Moses B, Orchard J, Orchard J. Systematic review: Annual incidence of ACL injury and surgery in various populations. *Res Sports Med.* 2012;20(3-4):157-179.
6. Werner BC, Yang S, Looney AM, Gwathmey FW, Jr. Trends in Pediatric and Adolescent Anterior Cruciate Ligament Injury and Reconstruction. *J Pediatr Orthop.* 2016;36(5):447-452.
7. Lohmander LS, Englund PM, Dahl LL, Roos EM. The long-term consequence of anterior cruciate ligament and meniscus injuries: osteoarthritis. *Am J Sports Med.* 2007;35(10):1756-1769.
8. Lohmander LS, Ostenberg A, Englund M, Roos H. High prevalence of knee osteoarthritis, pain, and functional limitations in female soccer players twelve years after anterior cruciate ligament injury. *Arthritis Rheum.* 2004;50(10):3145-3152.
9. Whittaker JL, Woodhouse LJ, Nettel-Aguirre A, Emery CA. Outcomes associated with early post-traumatic osteoarthritis and other negative health consequences 3-10 years following knee joint injury in youth sport. *Osteoarthritis Cartilage.* 2015;23(7):1122-1129.
10. Riordan EA, Frobell RB, Roemer FW, Hunter DJ. The health and structural consequences of acute knee injuries involving rupture of the anterior cruciate ligament. *Rheum Dis Clin North Am.* 2013;39(1):107-122.
11. Stiebel M, Miller LE, Block JE. Post-traumatic knee osteoarthritis in the young patient: therapeutic dilemmas and emerging technologies. *Open Access J Sports Med.* 2014;5:73-79.
12. Lie MM, Risberg MA, Storheim K, Engebretsen L, Oiestad BE. What's the rate of knee osteoarthritis 10 years after anterior cruciate ligament injury? An updated systematic review. *Br J Sports Med.* 2019;53(18):1162-1167.
13. Altman R, Asch E, Bloch D, et al. Development of criteria for the classification and reporting of osteoarthritis. Classification of osteoarthritis of the knee. Diagnostic and Therapeutic Criteria Committee of the American Rheumatism Association. *Arthritis Rheum.* 1986;29(8):1039-1049.
14. Felson DT. The epidemiology of knee osteoarthritis: results from the Framingham Osteoarthritis Study. *Semin Arthritis Rheum.* 1990;20(3 Suppl 1):42-50.
15. Horga LM, Hirschmann AC, Henckel J, et al. Prevalence of abnormal findings in 230 knees of asymptomatic adults using 3.0 T MRI. *Skeletal Radiol.* 2020.
16. Felson DT. Osteoarthritis as a disease of mechanics. *Osteoarthritis Cartilage.* 2013;21(1):10-15.
17. Andriacchi TP, Mundermann A. The role of ambulatory mechanics in the initiation and progression of knee osteoarthritis. *Curr Opin Rheumatol.* 2006;18(5):514-518.
18. Wellsandt E, Khandha A, Manal K, Axe MJ, Buchanan TS, Snyder-Mackler L. Predictors of knee joint loading after anterior cruciate ligament reconstruction. *J Orthop Res.* 2017;35(3):651-656.

19. Tsai LC, McLean S, Colletti PM, Powers CM. Greater muscle co-contraction results in increased tibiofemoral compressive forces in females who have undergone anterior cruciate ligament reconstruction. *J Orthop Res.* 2012;30(12):2007-2014.
20. Capin JJ, Khandha A, Zarzycki R, et al. Gait mechanics and tibiofemoral loading in men of the ACL-SPORTS randomized control trial. *J Orthop Res.* 2018;36(9):2364-2372.
21. Capin JJ, Khandha A, Zarzycki R, Manal K, Buchanan TS, Snyder-Mackler L. Gait Mechanics After ACL Reconstruction Differ According to Medial Meniscal Treatment. *J Bone Joint Surg Am.* 2018;100(14):1209-1216.
22. Gardinier ES, Manal K, Buchanan TS, Snyder-Mackler L. Altered loading in the injured knee after ACL rupture. *J Orthop Res.* 2013;31(3):458-464.
23. Slater LV, Hart JM, Kelly AR, Kuenze CM. Progressive Changes in Walking Kinematics and Kinetics After Anterior Cruciate Ligament Injury and Reconstruction: A Review and Meta-Analysis. *J Athl Train.* 2017;52(9):847-860.
24. Henriksen M, Creaby MW, Lund H, Juhl C, Christensen R. Is there a causal link between knee loading and knee osteoarthritis progression? A systematic review and meta-analysis of cohort studies and randomised trials. *BMJ Open.* 2014;4(7):e005368.
25. Henriksen M, Hunter DJ, Dam EB, et al. Is increased joint loading detrimental to obese patients with knee osteoarthritis? A secondary data analysis from a randomized trial. *Osteoarthritis Cartilage.* 2013;21(12):1865-1875.
26. Oiestad BE, Holm I, Risberg MA. Return to pivoting sport after ACL reconstruction: association with osteoarthritis and knee function at the 15-year follow-up. *Br J Sports Med.* 2018;52(18):1199-1204.
27. Alkjaer T, Simonsen EB, Jorgensen U, Dyhre-Poulsen P. Evaluation of the walking pattern in two types of patients with anterior cruciate ligament deficiency: copers and non-copers. *Eur J Appl Physiol.* 2003;89(3-4):301-308.
28. Alkjaer T, Simonsen EB, Peter Magnusson SP, Aagaard H, Dyhre-Poulsen P. Differences in the movement pattern of a forward lunge in two types of anterior cruciate ligament deficient patients: copers and non-copers. *Clin Biomech (Bristol, Avon).* 2002;17(8):586-593.
29. DeVita P, Aaboe J, Bartholdy C, Leonardis JM, Bliddal H, Henriksen M. Quadriceps-strengthening exercise and quadriceps and knee biomechanics during walking in knee osteoarthritis: A two-centre randomized controlled trial. *Clin Biomech (Bristol, Avon).* 2018;59:199-206.
30. Radin EL, Yang KH, Riegger C, Kish VL, O'Connor JJ. Relationship between lower limb dynamics and knee joint pain. *J Orthop Res.* 1991;9(3):398-405.
31. Culvenor AG, Ruhdorfer A, Juhl C, Eckstein F, Oiestad BE. Knee Extensor Strength and Risk of Structural, Symptomatic, and Functional Decline in Knee Osteoarthritis: A Systematic Review and Meta-Analysis. *Arthritis Care Res (Hoboken).* 2017;69(5):649-658.
32. de Jong SN, van Caspel DR, van Haeff MJ, Saris DB. Functional assessment and muscle strength before and after reconstruction of chronic anterior cruciate ligament lesions. *Arthroscopy.* 2007;23(1):21-28, 28 e21-23.
33. Birchmeier T, Lisee C, Kane K, Brazier B, Triplett A, Kuenze C. Quadriceps Muscle Size Following ACL Injury and Reconstruction: A Systematic Review. *J Orthop Res.* 2020;38(3):598-608.
34. Lisee C, Lepley AS, Birchmeier T, O'Hagan K, Kuenze C. Quadriceps Strength and Volitional Activation After Anterior Cruciate Ligament Reconstruction: A Systematic Review and Meta-analysis. *Sports Health.* 2019;11(2):163-179.
35. Astephen Wilson JL, Stanish WD, Hubley-Kozey CL. Asymptomatic and symptomatic individuals with the same radiographic evidence of knee osteoarthritis walk with different knee moments and muscle activity. *J Orthop Res.* 2017;35(8):1661-1670.

36. Hiemstra B, Keus F, Wetterslev J, Gluud C, van der Horst ICC. DEBATE-statistical analysis plans for observational studies. *BMC Med Res Methodol.* 2019;19(1):233.
37. Bhatt A. Protocol deviation and violation. *Perspect Clin Res.* 2012;3(3):117.
38. Jaric S. Muscle strength testing: use of normalisation for body size. *Sports Med.* 2002;32(10):615-631.
39. Kristianslund E, Krosshaug T, van den Bogert AJ. Effect of low pass filtering on joint moments from inverse dynamics: implications for injury prevention. *J Biomech.* 2012;45(4):666-671.
40. Seth A, Hicks JL, Uchida TK, et al. OpenSim: Simulating musculoskeletal dynamics and neuromuscular control to study human and animal movement. *PLoS Comput Biol.* 2018;14(7):e1006223.
41. Bedo BLS, Catelli DS, Lamontagne M, Santiago PRP. A custom musculoskeletal model for estimation of medial and lateral tibiofemoral contact forces during tasks with high knee and hip flexions. *Comput Methods Biomech Biomed Engin.* 2020;23(10):658-663.
42. Saxby DJ, Bryant AL, Modenese L, et al. Tibiofemoral Contact Forces in the Anterior Cruciate Ligament-Reconstructed Knee. *Med Sci Sports Exerc.* 2016;48(11):2195-2206.
43. Ehrig RM, Taylor WR, Duda GN, Heller MO. A survey of formal methods for determining the centre of rotation of ball joints. *J Biomech.* 2006;39(15):2798-2809.
44. Ehrig RM, Taylor WR, Duda GN, Heller MO. A survey of formal methods for determining functional joint axes. *J Biomech.* 2007;40(10):2150-2157.
45. Michaud B, Begon M. Two efficient static optimization algorithms that account for muscle-tendon equilibrium: approaching the constraint Jacobian via a constant or a cubic spline function. *Comput Methods Biomech Biomed Engin.* 2020:1-7.
46. Sritharan P, Lin YC, Pandy MG. Muscles that do not cross the knee contribute to the knee adduction moment and tibiofemoral compartment loading during gait. *J Orthop Res.* 2012;30(10):1586-1595.
47. Roos EM, Lohmander LS. The Knee injury and Osteoarthritis Outcome Score (KOOS): from joint injury to osteoarthritis. *Health Qual Life Outcomes.* 2003;1:64.
48. Irrgang JJ, Anderson AF, Boland AL, et al. Development and validation of the international knee documentation committee subjective knee form. *Am J Sports Med.* 2001;29(5):600-613.
49. Hawker GA, Davis AM, French MR, et al. Development and preliminary psychometric testing of a new OA pain measure--an OARSI/OMERACT initiative. *Osteoarthritis Cartilage.* 2008;16(4):409-414.
50. Tegner Y, Lysholm J. Rating systems in the evaluation of knee ligament injuries. *Clin Orthop Relat Res.* 1985(198):43-49.
51. Jorgensen TS, Hangaard S, Bliddal H, Henriksen M. Test-retest reliability of cuff pressure pain algometry in patients with knee osteoarthritis. *Clin Exp Rheumatol.* 2016;34(1):158.
52. Jespersen A, Dreyer L, Kendall S, et al. Computerized cuff pressure algometry: A new method to assess deep-tissue hypersensitivity in fibromyalgia. *Pain.* 2007;131(1-2):57-62.
53. Kellgren JH, Lawrence JS. Radiological assessment of osteo-arthrosis. *Ann Rheum Dis.* 1957;16(4):494-502.
54. Henriksen M, Rosager S, Aaboe J, Graven-Nielsen T, Bliddal H. Experimental knee pain reduces muscle strength. *J Pain.* 2011;12(4):460-467.

Details of musculoskeletal modeling and simulation

The experimental data collection was conducted in a motion capture laboratory equipped with 12 infrared cameras and two force plates. Thirty-nine reflective markers were placed onto the volunteers according to an extended version of the lower body Conventional Gait Model 2.4 [1] with an additional five markers on the torso (Fig. S1). Subsequently, participants performed walking and forward lunge trials while marker trajectories (100 Hz, Vicon Motion Systems Ltd, UK) and ground reaction forces (1000 Hz, OR-6, Advanced Mechanical Technology Inc., USA) were recorded (Vicon Nexus version 2.12). After gap-filling for marker trajectories, ground reaction forces and marker trajectories were filtered using a zero-lag Butterworth low pass filter (fourth order, 6 Hz cut-off frequency).

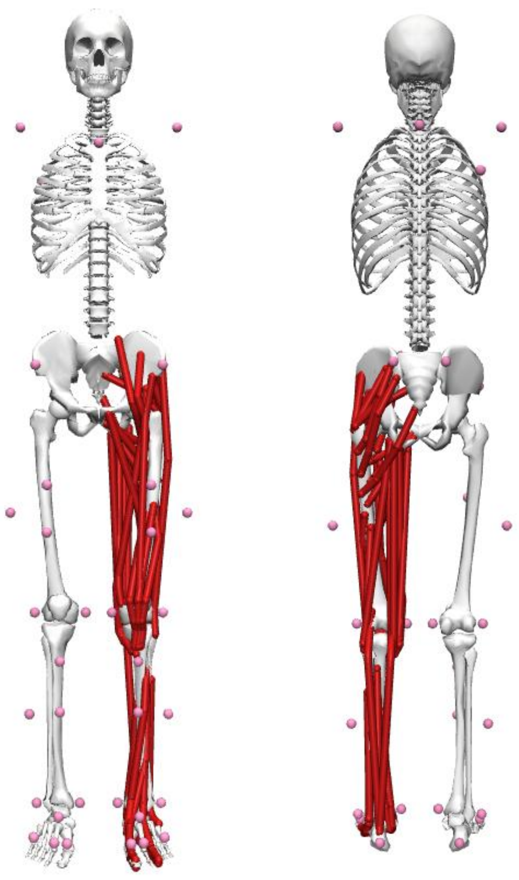

Figure S1. Musculoskeletal model and location of experimental motion capture markers. Note that the model shown is one used to simulate the left leg ground contact phase. The muscles of the contralateral side were removed to reduce simulation time and the joint on that side was actuated with reserve actuators. For simulation, the right leg ground contacts a similar model but with muscle-actuated joints on the right leg.

Musculoskeletal model

OpenSim (version 4.1) [2] was used for biomechanical modeling and simulation. We used a musculoskeletal model particularly developed for movements with large hip and knee flexion range of motion and the ability to separate the joint contact forces (JCFs) transmitted through the medial and lateral tibiofemoral joint [3]. The model had 3 degree-of-freedom (DoF) hip, 1 DoF knee, and 1 DoF ankle joint. Subtalar ankle was kept locked. We further modified the model's quadriceps moment arms to avoid overestimating the knee JCFs [4] by adjusting the moment arm to better fit a value proposed in the literature for vastus lateralis [5]. The moment arm modification was achieved by setting an individual wrapping surface for each quadriceps muscle and adjusting the coupled coordinate scaling factor of the patella which reduced the patella movement as a function of knee flexion angle. We also modified lower limb muscle properties of the reconstructed leg to reflect

adaptations reported in the literature in individuals with ACL reconstruction using semitendinosus-gracilis graft [6,7] (details below). Finally, we deleted one of the via-points of the iliacus and psoas muscles as we noted that in cases in which the pelvis was scaled proportionally larger than the femur the via point penetrated the wrapping surface of the respective muscles in large hip extension resulting in an abrupt shortening of the muscle-tendon length and subsequent simulation errors. The model is available online. https://simtk.org/projects/mirakos.

Modification of the muscle-tendon properties of the ACL-reconstructed leg

We created a separate model for simulating the stance phases of gait and lunge of the leg with ACL reconstruction. In this model, we modified the optimal fiber length, optimal force (i.e., maximal isometric muscle force at optimal length), and tendon slack length of semitendinosus and gracilis muscles to account for the autograft donor muscle impairments and biceps femoris long head, semimembranosus, and vastus medialis to account for adaptations reported to occur in these intact muscles in individuals with ACL reconstruction [6]. The optimal fiber length and optimal force were modified by multiplying the values of the unscaled template model with a scale factor derived from relative values of muscle cross-sectional area and volume of the reconstructed and contralateral legs per Saxby et al. [7]. The cross-sectional areas and volumes were taken from Konrath et al. [6]. Finally, tendon slack length was adjusted to preserve the slack length of the muscle-tendon unit.

The modeling and simulation workflow

Modeling and simulation were performed in OpenSim (version 4.1) [2] utilizing the standard algorithms of the software. The model was scaled to match the dimensions of the individual using hip, knee, and ankle joint centers estimated from star-arc and squatting tasks, respectively using SCoRE and SARA algorithms implemented in Vicon Nexus [8,9]. Intercondylar distance (i.e., the frontal plane distance between the contact points at the medial and lateral tibiofemoral joint) was set based on the mediolateral distance between the midpoints of femoral condyles measured from radiographs using an approach based on automatic anatomical landmark localization of bones [10,11]. Optimal forces of the muscles (representing muscle strength) were scaled relative to the mass of the participant with allometric scaling [12] as done previously [13,14]. We additionally increased the optimal forces with a factor of 1.5 to ensure that the model was able to produce the high joint moments measured in forward lunging (equation 1).

$$\text{Optimal force scaling factor} = 1.5 * \left(\frac{\text{SUBJECT MASS}}{\text{GENERIC MODEL MASS}}\right)^{\frac{2}{3}} \quad (1)$$

Subsequently, to account for the participant-specific muscle strength deficit of the ACL-reconstructed leg in knee flexion and extension often present in ACL reconstructed individuals [15], we adjusted the optimal forces of the model's quadriceps and hamstring muscles in the reconstructed limb with a scale factor (common for the muscle group) that replicated the experimentally measured interlimb difference in muscle strength (joint moment) at the same joint configuration in which the experimental test was performed while maximally activating the quadriceps/hamstring muscles.

The simulations were performed for the stance phases which were detected based on 10 N vertical ground reaction force threshold. Inverse kinematics was executed to find the generalized coordinates (i.e., joint angles) by minimizing the sum of squared differences between experimental and model markers. Inverse dynamics was used to calculate the net joint moments with the ground reactions forces and kinematics as inputs after filtering them with a 6 Hz low pass filter in OpenSim. Muscle forces were estimated using static optimization with an objective function that minimized the sum of squared muscle activations. The joint reaction analysis tool was used to extract the compressive tibiofemoral JCFs. Distractive forces (i.e., negative forces when the compressive direction is positive) reported either on the medial or lateral tibiofemoral compartment were considered unphysiological and caused by collateral ligaments opposing the joint distraction. When distractive forces were reported, the compartment force was set to zero and a force

representing the collateral ligament force was added to the tibiofemoral joint [16]. The force attributed to the collateral ligament was assumed to exert its influence at a distance equivalent to half the width of the femur, as measured from the radiographs, relative to the centre of the knee joint. The femoral width was measured using an automatic anatomical landmark localization of bones [10,11].

References

1. CGM 2.4 markerset. https://pycgm2.netlify.app/cgm/cgm2.4/ (2024).
2. Seth, A., Sherman, M., Reinbolt, J. A. & Delp, S. L. OpenSim: a musculoskeletal modeling and simulation framework for in silico investigations and exchange. *Procedia IUTAM* **2**, 212–232 (2011).
3. Bedo, B. L. S., Catelli, D. S., Lamontagne, M. & Santiago, P. R. P. A custom musculoskeletal model for estimation of medial and lateral tibiofemoral contact forces during tasks with high knee and hip flexions. *Comput Methods Biomech Biomed Engin* **23**, 658–663 (2020).
4. Bosch, W. *et al.* Alterations in the Functional Knee Alignment Are Not an Effective Strategy to Modify the Mediolateral Distribution of Knee Forces During Closed Kinetic Chain Exercises. *J Appl Biomech* **38**, 424–433 (2022).
5. Bakenecker, P., Raiteri, B. & Hahn, D. Patella tendon moment arm function considerations for human vastus lateralis force estimates. *J Biomech* (2019) doi:10.1016/j.jbiomech.2019.01.042.
6. Konrath, J. M. *et al.* Morphologic characteristics and strength of the hamstring muscles remain altered at 2 years after use of a hamstring tendon graft in anterior cruciate ligament reconstruction. *Am J Sports Med* **44**, 2589–2598 (2016).
7. Saxby, D. J. *et al.* Tibiofemoral contact forces in the anterior cruciate ligament-reconstructed knee. *Med Sci Sports Exerc* **48**, 2195–2206 (2016).
8. Ehrig, R., Taylor, W., Duda, G. & Heller, M. A survey of formal methods for determining the centre of rotation of ball joints. *J Biomech* **39**, 2798–2809 (2006).
9. Ehrig, R. M., Taylor, W. R., Duda, G. N. & Heller, M. O. A survey of formal methods for determining functional joint axes. *J Biomech* **40**, (2007).
10. Lindner, C., Thiagarajah, S., Wilkinson, J. M., Wallis, G. A. & Cootes, T. F. Fully automatic segmentation of the proximal femur using random forest regression voting. *IEEE Trans Med Imaging* **32**, 1462–1472 (2013).
11. Neumann, G. *et al.* Location specific radiographic joint space width for osteoarthritis progression. *Osteoarthritis Cartilage* **17**, 761–765 (2009).
12. Folland, J. P., Mc Cauley, T. M. & Williams, A. G. Allometric scaling of strength measurements to body size. *Eur J Appl Physiol* **102**, 739–745 (2008).
13. Van Der Krogt, M. M., Bar-On, L., Kindt, T., Desloovere, K. & Harlaar, J. Neuro-musculoskeletal simulation of instrumented contracture and spasticity assessment in children with cerebral palsy. *J Neuroeng Rehabil* **13**, 1–11 (2016).
14. Rabbi, M. F. *et al.* Muscle synergy-informed neuromusculoskeletal modelling to estimate knee contact forces in children with cerebral palsy. *Biomech Model Mechanobiol* 1–14 (2024) doi:10.1007/S10237-024-01825-7/TABLES/5.
15. Tayfur, B., Charuphongsa, C., Morrissey, D. & Miller, S. C. Neuromuscular Function of the Knee Joint Following Knee Injuries: Does It Ever Get Back to Normal? A Systematic Review with Meta-Analyses. *Sports Medicine* **51**, 321–338 (2021).
16. Winby, C. R., Lloyd, D. G., Besier, T. F. & Kirk, T. B. Muscle and external load contribution to knee joint contact loads during normal gait. *J Biomech* **42**, 2294–2300 (2009).
